# Oropharyngeal Interventions in Intubated Patients for Preventing Ventilator Associated Pneumonia: A Systematic Review and Multi-Variate Network Meta-Analysis Evaluating Pharmacological Agents

**DOI:** 10.3390/jcm14228174

**Published:** 2025-11-18

**Authors:** Kannan Sridharan, Gowri Sivaramakrishnan, Ghazi Abdulrahman Alotaibi

**Affiliations:** 1 Department of Pharmacology & Therapeutics, College of Medicine & Health Sciences, Arabian Gulf University, Manama 26671, Bahrain; 2 Bahrain Defence Force Royal Medical Services, Riffa 28743, Bahrain; 3 Arabian Gulf University, Manama 26671, Bahrain; 4 Imam Abdulrahman Bin Faisal University, Dammam 31441, Saudi Arabia

**Keywords:** chlorhexidine, povidone iodine, toothbrush, VAP, ICU

## Abstract

**Background:** Ventilator-associated pneumonia (VAP) is a prevalent and serious complication of invasive mechanical ventilation (MV), contributing to significant mortality and increased healthcare resource utilization. While numerous oropharyngeal interventions exist, their comparative efficacy across critical outcomes remains uncertain due to a lack of direct comparisons in clinical trials. **Methods:** We conducted a systematic review and network meta-analysis (NMA) with a comprehensive search of MEDLINE, EMBASE, and Cochrane CENTRAL up to September 2025 for randomized and non-randomized studies comparing topical oral interventions in intubated patients. The primary outcome was VAP incidence; secondary outcomes were intensive care unit (ICU) mortality, duration of MV, and ICU length of stay (LOS). Pairwise and network meta-analyses were performed, and the certainty of evidence was assessed. The effect estimates were odds ratios (OR) for categorical outcomes and mean difference (MD) for numerical outcomes represented with 95% confidence intervals (95% CI). **Results:** Ninety-six studies (20,650 patients) were included, evaluating 44 interventions. For VAP prevention, several interventions were superior to reference/control, including Antimicrobial combinations (OR: 0.21, 95% CI: 0.05–0.39), Povidone-iodine (OR: 0.47, 95% CI: 0.21–0.98), and Chlorhexidine (OR 0.61, 95% CI 0.39–0.95). However, only Chlorhexidine plus toothbrushing significantly reduced mortality (OR: 0.74, 95% CI: 0.58–0.93). For resource utilization, only antimicrobial combinations significantly reduced the duration of MV (MD: −5.55 days, 95% CI: −10.75–−1.7) and ICU LOS (MD: −7.74 days, 95% CI: −13–−4). Evidence certainty (GRADE) was moderate for chlorhexidine and very low for other comparisons. **Conclusions:** This NMA demonstrates that while multiple oropharyngeal interventions are effective for VAP prevention, their benefits are outcome specific. The choice of intervention should be guided by clinical priorities, as the most effective strategy for preventing VAP may not concurrently reduce mortality or resource use. These findings can inform guideline development and underscore the need for standardized, multi-faceted oral care protocols in the ICU.

## 1. Introduction

Invasive mechanical ventilation (IMV) is a cornerstone of life support in the intensive care unit (ICU), yet it carries significant iatrogenic risks, most notably ventilator-associated pneumonia (VAP) [1]. VAP, defined as a lung parenchymal infection occurring more than 48 h after endotracheal intubation, represents a prevalent and serious complication. Its incidence is reported to range between 8–28%, equating to 1.4–16.5 episodes per 1000 ventilator days, making it the most common nosocomial infection among critically ill patients [2,3]. The clinical and economic burdens of VAP are substantial, contributing to an attributable mortality of approximately 10% (with even higher estimates in surgical ICUs), prolonged durations of mechanical ventilation, extended ICU and hospital stays, and significantly increased healthcare costs [4]. Microbiological analyses indicate that the most common pathogens responsible for VAP include *Pseudomonas species* (21%), *Staphylococcus aureus* (20.2%), and *Klebsiella species* (20.1%), with patient-specific risk factors such as advanced age and pre-existing chronic liver or kidney disease further compounding the risk [5].

In response to this significant challenge, multifaceted preventive strategies have been developed, primarily operationalized as “VAP bundles.” These bundles incorporate a suite of evidence-based interventions, including strict hand hygiene, daily sedation holidays and spontaneous breathing trials, elevation of the head of the bed to 30–45 degrees, and prophylaxis for stress ulcers and deep vein thrombosis [6]. The systematic implementation of such bundles has proven highly effective; a systematic review of 38 studies demonstrated a 36% reduction in VAP incidence, with nearly a third of the included studies reporting a decrease exceeding 65% [7].

A critical component within these preventive bundles is oropharyngeal care. The oral cavity is naturally protected by a sophisticated host defense system comprising physical barriers, immunological factors in saliva and gingival crevicular fluid, and a balanced oral microbiome [8]. However, critical illness and the requisite invasive interventions in the ICU profoundly compromise these defenses. Patients often experience xerostomia, impaired mucosal integrity, and a shift in oral ecology toward pathogenic colonization. This dysbiosis can transform the oropharynx into a reservoir for pathogens, facilitating their microaspiration around the endotracheal tube cuff, which can lead to systemic dissemination, bacteremia, and ultimately, VAP [9].

Consequently, a variety of oropharyngeal decontamination and hygiene interventions have been investigated to mitigate this risk. These include mechanical cleansing with toothbrushing and the application of topical pharmacological agents such as chlorhexidine gluconate and povidone-iodine. Furthermore, selective oral decontamination (SOD) with non-absorbable antimicrobial pastes or gels, containing agents like colistin, tobramycin, gentamicin, vancomycin, and amphotericin B, has been explored with variable efficacy [10]. Herbal preparations, such as those based on ginger, cinnamon, or other botanicals with purported antimicrobial and anti-inflammatory properties, have also been studied as potential alternatives. For this study, we systematically evaluated the following categories of oropharyngeal interventions against each other and against standard care or placebo: (1) mechanical hygiene (toothbrushing), (2) topical antiseptics (such as chlorhexidine, povidone-iodine), (3) selective oral decontamination (SOD) with antibiotic/antifungal pastes, and (4) herbal-based formulations. Despite its established importance, oropharyngeal care in intubated patients remains inconsistently standardized, with ongoing debate regarding the optimal choice of agent, frequency, and protocol [11]. Toothbrushing is the only recommended as the only intervention according to the expert panel convened by the International Society of Infectious Diseases [12].

The evidence for these diverse interventions has been synthesized in several conventional pairwise meta-analyses [13,14,15,16]. However, these syntheses are constrained by several limitations. First, they often fail to compare the full spectrum of available interventions, encompassing both allopathic and herbal products, on a single, unified platform. Second, many lack a thorough assessment of the certainty (or strength) of the evidence, such as through the GRADE (Grading of Recommendations, Assessment, Development, and Evaluations) framework. Finally, the primary obstacle to direct comparison is the scarcity of head-to-head randomized controlled trials (RCTs) comparing all relevant interventions.

Network meta-analysis (NMA) offers a powerful statistical methodology to overcome this evidence gap. By integrating direct evidence (from head-to-head trials) and indirect evidence (derived through common comparator interventions), NMA permits concurrent comparison (and ranking) of multiple interventions, despite the lack of direct comparative trials [17]. Therefore, this study aims to employ a multivariate network meta-analysis to comprehensively evaluate and rank the efficacy of all pertinent oropharyngeal interventions for the prevention of VAP in intubated patients. Our primary objective is to compare their relative effects on the incidence of VAP, with secondary outcomes including all-cause mortality, ICU length of stay, and duration of invasive mechanical ventilation.

## 2. Methods

### 2.1. Search Methods

The protocol for this review was registered with the Open Science Framework [18]. Following databases were searched for eligible studies: MEDLINE (via PubMed), EMBASE, and the Cochrane Central Register of Controlled Trials (CENTRAL), from their inception until 24 September 2025, to identify studies evaluating topical oropharyngeal interventions in intubated patients. The search strategy utilized a combination of Medical Subject Headings (MeSH) and free-text terms related to both the interventions (e.g., chlorhexidine, toothbrushing, antiseptics, antibiotics, herbal extracts) and the context (e.g., mechanical ventilation, ventilator-associated pneumonia). The full search strategy for each database is provided in Electronic Supplementary Table S1. As an example, the PubMed search string is detailed in the introduction. No restrictions were placed on language or publication dates to maximize inclusivity. Conference abstracts were excluded due to typically insufficient methodological and outcome details, which would preclude a robust assessment of risk of bias.

### 2.2. Eligibility Criteria

Both RCTs and observational studies were considered eligible for inclusion. The inclusion criteria were as follows:

Population: Children and adult patients receiving invasive mechanical ventilation.
Interventions: Any topical oral intervention aimed at preventing VAP such as Chlorhexidine (various concentrations: 0.12%, 0.2%, 1%, 2%), Povidone-iodine, Probiotics (such as *Lactobacillus*), Antimicrobial drugs (such as polymyxin, tobramycin), Iseganan, Silver nanoparticles, Hydrogen peroxide, Sodium bicarbonate, Toothbrush, Potassium permanganate, Ozonated water, Nanosil, Miswak, Triclosan, Listerine, Nitrofurazone, Biotene, Amphotericin B, Chinese herbal formulation, Persica, Matrica, Achillea millefolium, Mentha spicata, Chamomile.
Comparators: Standard of care, placebo, water, saline or any of the above interventions.
Outcomes: The primary outcome was the incidence of VAP. We considered any definition used by the original study authors, such as clinical pulmonary infection score (CPIS), Centers for Disease Control and Prevention (CDC), American Thoracic Society/Infectious Disease Society of America, Chinese Society for Respiratory Disease or bacteriologic confirmation. The secondary outcomes were all-cause ICU mortality, duration of mechanical ventilation and ICU stay.

### 2.3. Study Selection and Data Extraction

Two authors were involved in the independent screening of the retrieved records based on titles and abstracts, and in the full-text evaluation of potentially eligible articles. Any discrepancy was resolved through a consensus or discussion with the third author. From each included study, the following data were extracted: study characteristics: first author, year, country, setting, design; participant characteristics: number randomized, age, gender, APACHE/SAPS score, ICU type; intervention and comparator details: specific drug, concentration, frequency, delivery method, co-interventions (toothbrushing); and outcomes.

### 2.4. Data Synthesis and Statistical Analysis

The methodological quality of included RCTs was assessed using the Cochrane risk of bias tool [19]. For observational and non-randomized studies, the Joanna Briggs Institute (JBI) critical appraisal checklist was used [20].

Quantitative synthesis was performed using a frequentist framework for network meta-analysis (NMA), employing a mixed-treatment comparison model to integrate direct and indirect evidence. Effect sizes were expressed as Odds Ratios (OR) with 95% Confidence Intervals (CI) for categorical outcomes and Mean Differences (MD) with 95% CI for numerical outcomes. The reference treatment group for comparisons comprised control interventions such as placebo, saline, water, standard of care, or no oral care. Heterogeneity was quantified using the I^2^ statistic, with values interpreted as follows: 0–25% (low), 25–50% (moderate), 50–75% (substantial), and 75–100% (considerable) [19]. The relative ranking of interventions for each outcome was estimated using the Surface Under the Cumulative Ranking curve (SUCRA). To assess the robustness of the findings, we performed a bootstrap analysis (1000 iterations) and a leave-one-out sensitivity analysis for key interventions (Chlorhexidine, Chlorhexidine + Toothbrush, Antimicrobial combinations, and Povidone iodine) compared to the reference.

For outcomes with significant pooled estimates in pairwise meta-analyses, we conducted cumulative meta-analysis and Trial Sequential Analysis (TSA) to evaluate the reliability of the evidence. Pre-specified subgroup analyses included: study design (RCTs only), chlorhexidine concentration, the presence or absence of toothbrushing, VAP definition, and VAP bundle adequacy. VAP bundle adequacy was scored by awarding one point for each of the following co-interventions: daily sedation interruption, stress ulcer prophylaxis, deep vein thrombosis prophylaxis, head-of-bed elevation to 30–45°, use of a subglottic suctioning endotracheal tube, and regular endotracheal cuff pressure monitoring. The total score was categorized as: 0 (inadequate), 1 (basic), 2 (moderate), 3 (good), or 4 (comprehensive). Meta-regression was performed to obtain covariate-adjusted pooled estimates, with covariates including the specific oral care intervention, mean age, percentage of males, daily frequency of the intervention, and mean APACHE II score.

Publication bias was assessed using Egger’s regression test (*p* ≤ 0.05 indicate potential publication bias) and funnel plots for comparisons with adequate studies. The certainty of the evidence for key estimates was evaluated using the GRADE framework [19].

### 2.5. Supplementary Propensity Score Analysis

To complement the primary NMA and address potential confounding in non-randomized comparisons, we conducted a propensity score matching (PSM) analysis focusing on the comparative effectiveness of Chlorhexidine versus reference interventions on VAP rates. This analysis utilized study-level aggregate data from arms included in the NMA dataset. Propensity scores were estimated using logistic regression, with mean participant age, percentage of male participants, and mean baseline illness severity score as covariates. We employed 1:1 optimal matching with a caliper width of 0.2 standard deviations of the logit propensity score, which achieved the best covariate balance. Post-matching balance was assessed using standardized mean differences (SMD), with an SMD <0.2 indicating adequate balance. Outcomes were compared between the matched groups using Welch’s *t*-test, and sensitivity analyses were performed using regression adjustment on the matched sample. All PSM analyses were performed using R version 4.5.1.

This systematic review follows the Preferred Reporting Items for Systematic Reviews and Meta-Analyses (PRISMA) guidelines and the PRISMA checklist is available as Electronic Supplementary File S1 [21].

## 3. Results

### 3.1. Search Results

**Figure 1:**
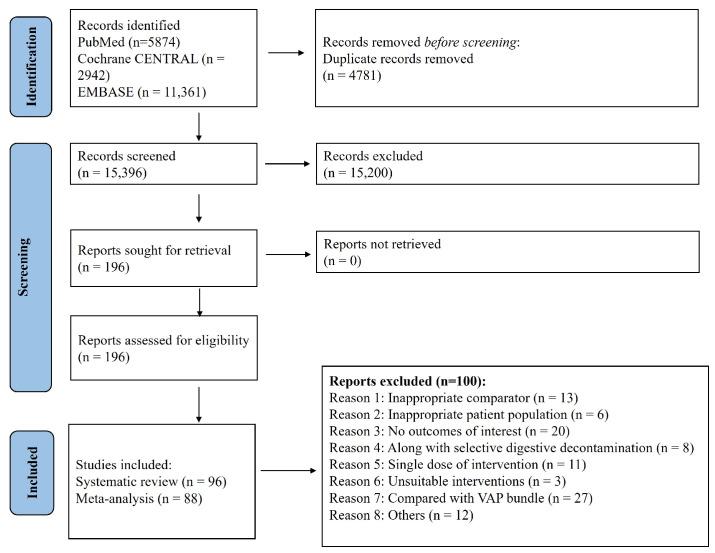
PRISMA flow diagram. A total of 96 studies were included in this systematic review and 88 in the meta-analysis.

A total of 96 studies (20,650 patients) [22,23,24,25,26,27,28,29,30,31,32,33,34,35,36,37,38,39,40,41,42,43,44,45,46,47,48,49,50,51,52,53,54,55,56,57,58,59,60,61,62,63,64,65,66,67,68,69,70,71,72,73,74,75,76,77,78,79,80,81,82,83,84,85,86,87,88,89,90,91,92,93,94,95,96,97,98,99,100,101,102,103,104,105,106,107,108,109,110,111,112,113,114,115,116,117] were included in the systematic review as depicted in the PRISMA flow diagram in Figure 1. Table 1 summarizes study characteristics by intervention type. The studies were primarily RCTs and were conducted across a wide range of countries, with a notable concentration of research from Iran. The ICU settings were varied, including medical, surgical, mixed, and cardiothoracic units. Patient severity at baseline was frequently assessed using the APACHE II score, with reported means typically ranging from the mid-teens to the low twenties. The mean age of enrolled patients was most reported to be between 40 and 65 years, and the proportion of male participants often varied from approximately 50% to 70%. The primary interventions investigated involved different forms of oral care, with Chlorhexidine at various concentrations being the most frequently studied agent, either used alone or in combination with toothbrushing. The definition of VAP also varied, with common criteria including the CPIS and CDC guidelines.

Eight studies did not report the values of the outcomes of interest in this study in a way amenable to statistical analysis due to which a total of 88 studies (20,131 patients) to be included in the meta-analysis. Amongst the studies included in the meta-analysis they were published between 1990 and 2025 across 24 countries, with the highest contributions from Iran (n = 25) followed by China (n = 10). The studies encompassed 189 study arms evaluating 44 unique oral care interventions. Many trials were conducted in mixed ICU settings (35.4%), followed by unclear settings (20.2%) and surgical ICUs (14.6%). Most studies employed RCT designs (79.5%), with additional contributions from cohort studies (18.2%) and non-randomized trials (2.3%). Follow-up duration primarily extended until hospital discharge or death (47.2% of studies), while VAP bundle adequacy scores ranged from 0 to 4 across the included trials. The intervention spectrum was dominated by Chlorhexidine-based regimens, with Chlorhexidine alone investigated in 54 studies (61.4%) and Chlorhexidine combined with Toothbrushing in 23 studies (26.1%). Toothbrush incorporation was reported in 46 study arms (24.3%), while 35 studies (39.8%) employed reference/control interventions. Amongst the Antimicrobial combinations (n = 5), Amphotericin B, Tobramycin and Polymyxin E were reported in three studies, Gentamicin, Colistin and Vancomycin in two and Polymyxin B, Neomycin and Vancomycin in one study. Amongst the standalone topical antimicrobial drug (n = 2), Metronidazole and Gentamicin were used in each. Outcome reporting was comprehensive for VAP (81 studies, 92% across 23 countries), though less complete for mortality (44 studies, 50% across 17 countries), duration of mechanical ventilation (40 studies, 45.5% across 17 countries), and ICU length of stay (38 studies, 43.2% across 17 countries), reflecting the primary focus on VAP prevention as the central outcome measure across this evidence base.

### 3.2. Network Meta-Analysis

**Figure 2:**
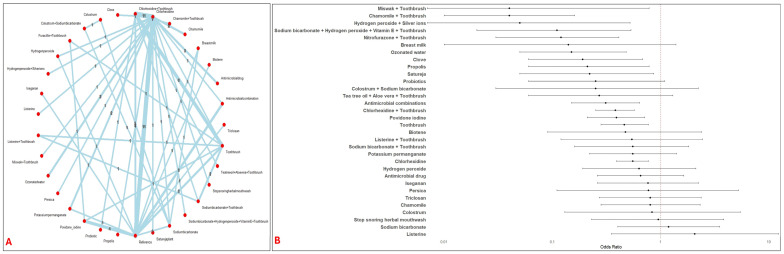
Network and Forest plots of oral interventions for VAP prevention. Figure 2 shows the network structure, and the effect estimates from the network meta-analysis of topical interventions for VAP prevention. Panel (**A**) (Network of Interventions for VAP Prevention) illustrates the geometry of the network. Panel (**B**) (Forest Plot VAP Prevention) displays the OR and 95% CI for each intervention compared to the reference intervention.

Eighty-one studies evaluating 34 distinct oral care interventions (15,117 patients) for the prevention of VAP in critically ill patients. The network was well-connected with adequate direct and indirect comparisons with majority of studies comparing chlorhexidine (Figure 2A). The following interventions demonstrated superior efficacy in terms of reduced risk of VAP occurrence compared to the reference interventions: Miswak + Toothbrush (OR: 0.04; 95% CI: 0–0.78), Chamomile + Toothbrush (OR: 0.04; 95% CI: 0.01, 0.16), Hydrogen peroxide + Silver ions (OR: 0.05; 95% CI: 0, 0.52), Hydrogen peroxide + Vitamin E + Toothbrush (OR: 0.11; 95% CI: 0.02, 0.53), Nitrofurazone + Toothbrush (OR: 0.12; 95% CI: 0.03, 0.41), Ozonated water (OR: 0.15; 95% CI: 0.05, 0.48), Clove (OR: 0.19; 95% CI: 0.6, 0.68), Propolis (OR: 0.21; 95% CI: 0.06, 0.78), Satureja plant (OR: 0.22; 95% CI: 0.05, 0.86), Antimicrobial combination (OR: 0.31; 95% CI: 0.15, 0.64), Chlorhexidine (OR: 0.55; 95% CI: 0.39, 0.75), Chlorhexidine + Toothbrush (OR: 0.38; 95% CI: 0.25, 0.57), Povidone iodine (OR: 0.39; 95% CI: 0.21, 0.71)and Toothbrush alone (OR: 0.46; 95% CI: 0.28, 0.77) (Figure 2B). A substantial heterogeneity was observed (I^2^ = 61.1%).

**Figure 3:**
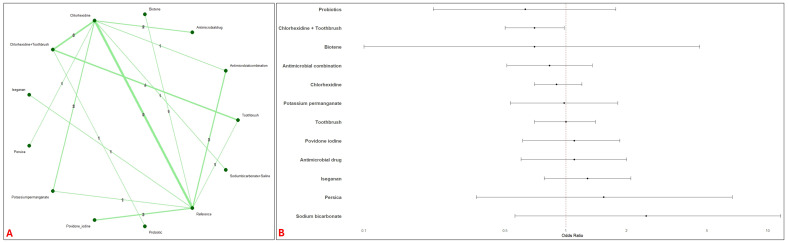
Network and Forest plots of oral interventions for the risk of mortality. This figure illustrates the network structure and effect estimates from the network meta-analysis of topical oral interventions on mortality risk. Panel (**A**) (Network of Interventions for Mortality) displays the geometry of the network. Panel (**B**) (Forest Plot Mortality—All Interventions vs. Reference) displays the OR and 95% CI for each intervention compared to the reference intervention.

Forty-four studies evaluating 13 interventions (12,433 patients) were included for the assessment of mortality risk. The network had adequate direct and indirect comparisons with majority of studies comparing Chlorhexidine (Figure 3A). No interventions were significantly associated with reduced risk of mortality except Chlorhexidine + Toothbrush (OR: 0.74; 95% CI: 0.58, 0.93) (Figure 3B). A low heterogeneity was observed (I^2^ = 19%).

**Figure 4:**
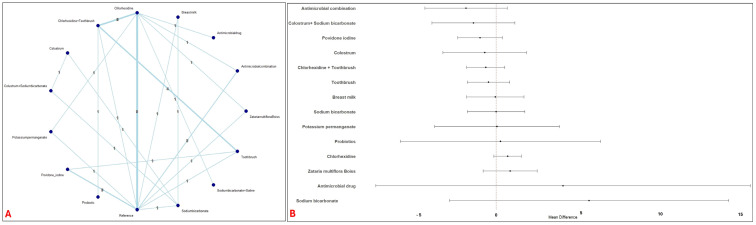
Network and Forest plots of oral interventions for the duration of mechanical ventilation. This figure displays the network structure and effect estimates from the network meta-analysis concerning the duration of mechanical ventilation. Panel (**A**) (Network of interventions for mechanical ventilation duration) illustrates the geometry of the network. Panel (**B**) (Forest Plot mechanical ventilation duration) displays the MD and 95% CI for each intervention compared to the reference intervention.

Thirty-nine studies evaluating 15 interventions (7236 patients) were included for the assessment of duration of mechanical ventilation. The network had adequate direct and indirect comparisons with majority of studies comparing Chlorhexidine (Figure 4A). No significant differences were observed with any of the interventions (Figure 4B). A moderate heterogeneity was observed (I^2^ = 45.6%).

**Figure 5:**
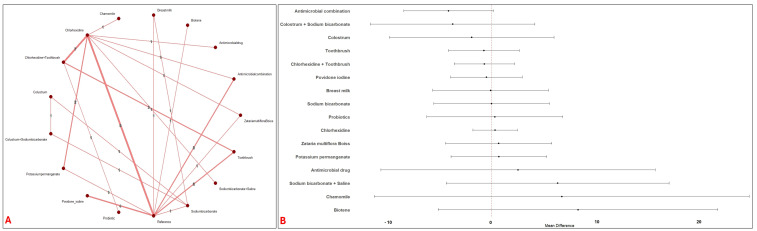
Network and Forest plots of oral interventions for the duration of stay in ICU. The figure displays the results of a network meta-analysis examining the effect of various interventions on ICU stay duration. Panel (**A**) is the network of interventions for ICU stay duration, which illustrates the comparisons made between different treatments. Panel (**B**) is the forest plot showing the mean difference in ICU stay duration for each intervention compared to a common reference group.

Thirty-nine studies evaluating 17 interventions (8956 patients) were included for the assessment of duration of ICU stay. The network had adequate direct and indirect comparisons with majority of studies comparing Chlorhexidine (Figure 5A). No significant differences were observed with any of the interventions in random effects model (Figure 5B) although Antimicrobial combination (MD: −4.13; 95% CI: −6.54 to −1.72), Colostrum + Sodium bicarbonate (MD: −3.73; 95% CI: −4.57 to −2.89), Chlorhexidine + Toothbrush (MD: −3.05; 95% CI: −4.11 to −1.99) and Colostrum (MD: −1.9; 95% CI: −2.69 to −1.11) were observed with significantly shorter ICU stay in fixed-effects model. A considerable heterogeneity was observed (I^2^ = 80.4%).

### 3.3. Ranking of Treatments by SUCRA Plots

**Figure 6:**
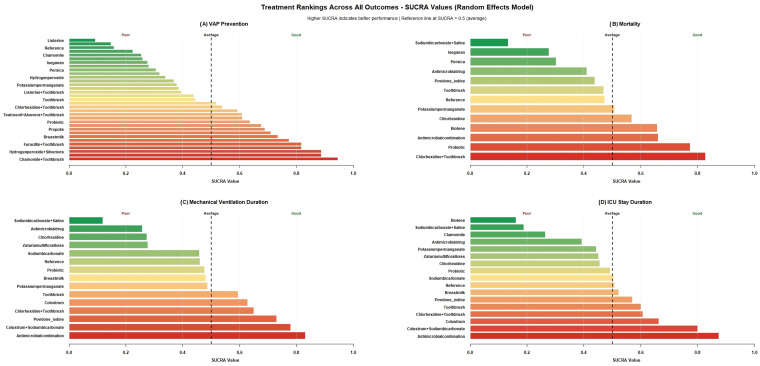
Comprehensive SUCRA plot for treatment ranking. This figure displays the treatment rankings across all outcomes from the network meta-analysis using the SUCRA values derived from the random effects Model. Panel (**A**) ranks interventions for VAP prevention. Panel (**B**) ranks interventions for mortality. Panel (**C**) ranks interventions for mechanical ventilation duration. Panel (**D**) ranks interventions for ICU stay duration.

The cumulative ranking probabilities, summarized by the SUCRA values, are presented in Figure 6. The analysis revealed that no single intervention consistently ranked best across all four: VAP prevention, mortality, duration of mechanical ventilation, and ICU length of stay. However, Antimicrobial combination ranked the best for durations of mechanical ventilation and ICU stay, and third for mortality risk reduction. Along with Toothbrush, Chamomile ranked the best for VAP prevention, and Chlorhexidine for mortality risk reduction. This outcome-specific performance highlights the trade-offs clinicians must consider when selecting a VAP prevention strategy, as the optimal choice may vary depending on the primary clinical goal.

### 3.4. Pairwise Meta-Analysis with Reference Intervention

**Figure 7:**
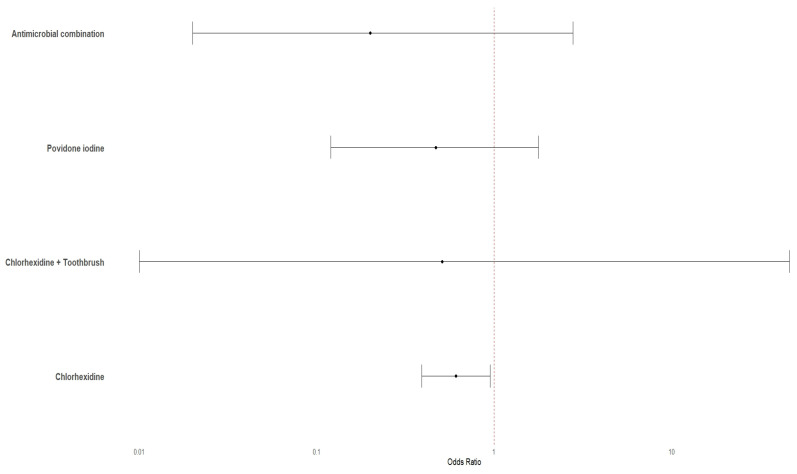
Pooled odds ratios for VAP prevention interventions. This forest plot displays the pooled odds ratios (OR) with 95% confidence intervals (CI) for different interventions aimed at VAP prevention, compared to a reference intervention.

Only four interventions were compared to reference intervention in head-to-head clinical trials that are amenable to direct comparison meta-analysis. In the pairwise meta-analyses comparing various interventions to reference, the only statistically significant reduction in odds was observed for Chlorhexidine (OR 0.61, 95% CI 0.39 to 0.95, *p* = 0.03). The point estimates for povidone iodine (OR 0.47) and the combination of Chlorhexidine + Toothbrush (OR 0.51) also suggested potential benefit, but their 95% confidence intervals were wide and included the null value (0.12 to 1.78 and 0.01 to 46, respectively), rendering them statistically non-significant (Figure 7). Similarly, the analysis for Antimicrobial combination (OR 0.21) was not statistically significant, with a confidence interval spanning from 0.02 to 2.78. Heterogeneity was consistently low across all comparisons (I^2^ <0.7%).

For mortality outcomes, none of the investigated interventions demonstrated a statistically significant effect. The odds ratios for antimicrobial combination (OR 0.75), povidone iodine (OR 0.97), and chlorhexidine (OR 0.94) all had 95% confidence intervals that included the null value, with ranges of 0.24 to 2.3, 0.21 to 4.45, and 0.73 to 1.2, respectively. The *p*-values for these comparisons were 0.38, 0.93, and 0.55, confirming the lack of statistical significance, and heterogeneity was negligible across all analyses (I^2^ ≤ 0.3%).

Regarding the outcome on mechanical ventilation duration, none of the interventions showed a statistically significant effect. The mean difference for Antimicrobial combination was −5.55 days (95% CI −62.41 to 51.31, *p* = 0.43), for Povidone iodine was −0.14 days (95% CI −0.98 to 0.69, *p* = 0.54), and for Chlorhexidine was 0.42 days (95% CI −0.04 to 0.88, *p* = 0.06). All confidence intervals crossed the null value of zero, indicating no conclusive evidence that any intervention meaningfully alters the duration of mechanical ventilation compared to the reference, with consistently low heterogeneity (I^2^ ≤ 0.8%) across all comparisons.

In the pairwise meta-analyses of ICU length of stay, none of the interventions demonstrated a statistically significant effect on duration. The mean difference for Antimicrobial combination was −7.74 days (95% CI −64.10 to 48.62, *p* = 0.33), for Povidone iodine was 0.22 days (95% CI −0.43 to 0.86, *p* = 0.37), and for Chlorhexidine was 1.04 days (95% CI −3.65 to 5.73, *p* = 0.61). The confidence intervals for all three comparisons were wide and included the null value of zero, indicating no conclusive evidence that any intervention meaningfully alters ICU stay duration compared to the reference, with heterogeneity consistently low across all analyses (I^2^ ≤ 0.9%).

### 3.5. Bootstrap Analyses

The bootstrap analysis, providing robust estimates of effect sizes and their confidence intervals, revealed distinct performance profiles across the interventions for the four primary outcomes (Electronic Supplementary Figure S1). For the prevention of VAP, the Antimicrobial combination was the most effective intervention, significantly reducing the odds of VAP (OR 0.21, 95% CI [0.05, 0.39]), followed by Povidone iodine (OR 0.47, 95% CI [0.21, 0.98]). In contrast, none of the interventions demonstrated a statistically significant effect on mortality reduction. Regarding the duration of mechanical ventilation, only the Antimicrobial combination showed a significant benefit, substantially reducing ventilation time by a mean of −5.55 days (95% CI [−10.75, −1.7]). This advantage extended to the ICU length of stay, where the Antimicrobial combination was again the sole intervention associated with a statistically significant reduction, shortening the stay by −7.742 days (95% CI [−13, −4]). Chlorhexidine-based interventions did not show significant benefits for mortality, ventilation duration, or ICU stay.

### 3.6. Sub-Group Analyses

The forest plots of interventions in various sub-groups are depicted in Electronic Supplementary Figure S2.

Amongst the studies with RCT design, Antimicrobial combinations, Chlorhexidine, Chlorhexidine + Toothbrush, Povidone iodine + Toothbrush and Toothbrush were observed to reduce VAP incidence; *Zataria multiflora Boiss.*+ Chlorhexidine significantly reduced the mortality risk; and none were observed to reduce either the duration of mechanical ventilation or ICU stay (Electronic Supplementary Figure S2A–D).

Amongst the varied definitions of VAP used, no significant differences were observed between the interventions on the outcomes except a reduction in the VAP incidence with Chlorhexidine and Antimicrobial combination when VAP is diagnosed according to CPIS (Electronic Supplementary Figure S2E).

Regarding the adequacy of VAP bundles incorporated, no significant differences were observed except a reduction in VAP incidence with antimicrobial combination in patients with basic bundle and Chlorhexidine + Toothbrush in patients without any VAP bundle (Electronic Supplementary Figure S2F).

Regarding the varied Chlorhexidine concentrations, significantly reduced duration of stay in ICU and mechanical ventilation were observed with 0.01–0.1% concentrations while 1.1–2% was observed with an increase in the duration of mechanical ventilation duration (Electronic Supplementary Figure S2G).

Regarding the concomitant use of Toothbrush, due to lack of adequate studies comparing with reference interventions, we could not estimate the pooled estimates for those using Toothbrush. However, amongst the studies without Toothbrush, Antimicrobial combination, Chlorhexidine, Hydrogen peroxide and Povidone iodine were observed with a reduction in the VAP incidence (Electronic Supplementary Figure S2H).

### 3.7. Multivariate Meta-Regression Analysis

**Figure 8:**
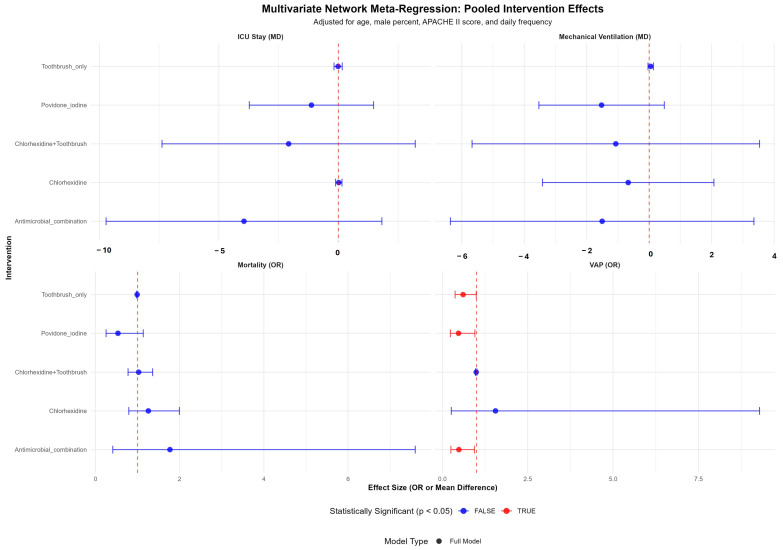
Facet plot for pooled estimates following multivariate meta-regression. The outcomes are displayed in four separate forest plots: ICU stay (mean difference), mechanical ventilation (mean difference), mortality (odds ratio), and VAP (odds ratio). The vertical dashed red lines represent the line of no effect. Each row represents a specific Intervention. Red data points indicate the effect is statistically Significant (*p* ≤ 0.05), while blue data points indicate the effect is not statistically significant.

The results of the multivariate network meta-regression showing the pooled intervention effects for four clinical outcomes, adjusted for covariates including age, percent male, APACHE II score, and daily frequency of the interventions are depicted in Figure 8. After the adjustment, Povidone iodine, Antimicrobial combination and Toothbrush were observed with significantly lower risk of VAP.

### 3.8. Publication Bias, Risk of Bias and Leave-One-Out Sensitivity Analysis

**Figure 9:**
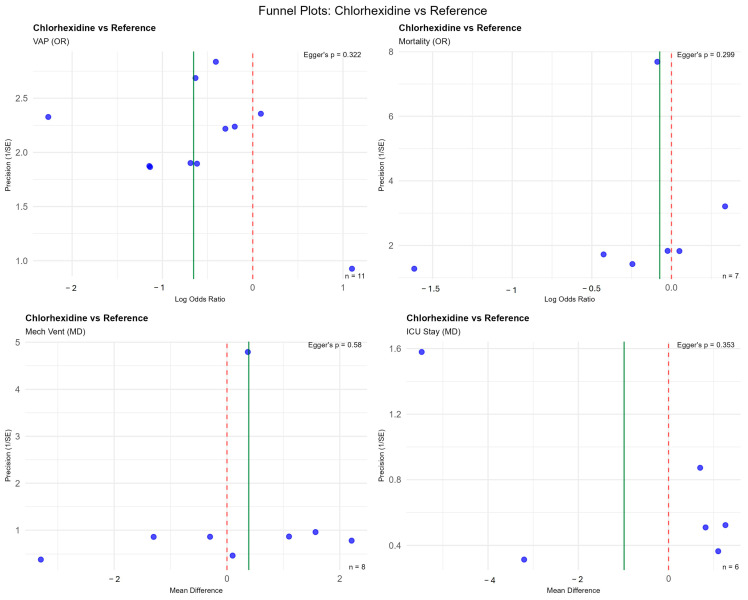
Funnel plots for assessing publication bias for chlorhexidine. Each plot presents the precision (calculated as standard error) on the y-axis against the effect size (log odds ratio or mean difference) on the x-axis. The data points represent individual studies included in the meta-analysis. The vertical dashed red line marks the null effect, and the solid green line represents the pooled effect estimate.

Publication bias could be assessed only for Chlorhexidine with reference interventions due to paucity of number of studies for other comparisons. No publication bias was evident both by funnel plots and Egger’s regression analyses (Figure 9).

**Figure 10:**
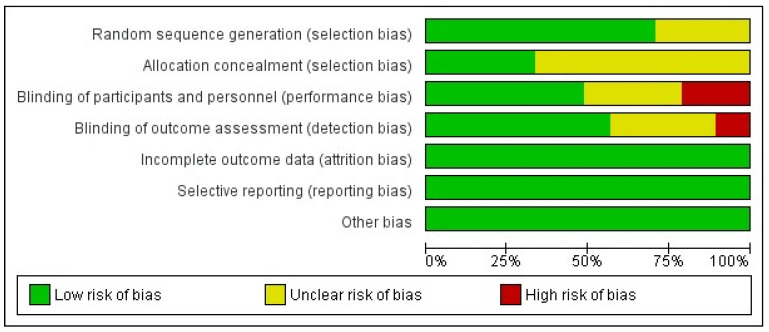
Risk of bias assessment of RCTs.

The risk of bias assessment of RCTs revealed an overall low risk (Figure 10). Similarly, the cohort studies and the non-randomized study were determined to have moderate quality.

Leave-one-out sensitivity analysis confirmed the robustness of the pooled estimates for the reduced risk of VAP with chlorhexidine (Electronic Supplementary Table S2) with varied results for other outcomes (Electronic Supplementary Tables S3–S5).

### 3.9. Cumulative Meta-Analysis and Trial Sequential Analysis

The cumulative meta-analysis for VAP prevention demonstrated a clear hierarchy in the efficacy of the assessed oral care interventions. The Antimicrobial combination emerged as the most potent intervention, yielding a statistically significant and substantial 78.6% reduction in the odds of VAP (OR 0.21, 95% CI: 0.07–0.65; *p* = 0.0065). Chlorhexidine alone also demonstrated a significant protective effect, associated with a 39.5% reduction in the odds of VAP (OR 0.61, 95% CI: 0.40–0.91; *p* = 0.0145). In contrast, Povidone iodine (OR 0.47, 95% CI: 0.21–1.06; *p* = 0.0683) and Chlorhexidine combined with toothbrushing (OR 0.51, 95% CI: 0.26–1.02; *p* = 0.0583) showed non-significant trends towards reducing VAP incidence (Electronic Supplementary Figures S3–S6).

Trial Sequential Analysis was conducted to evaluate the robustness and conclusiveness of the evidence for VAP prevention Both Chlorhexidine (final OR 0.58, Z-score −4.607) and the Antimicrobial combination (final OR 0.25, Z-score −4.832) demonstrated statistically robust, conclusive benefits, with their cumulative Z-curves crossing the trial sequential monitoring boundary for benefit (Electronic Supplementary Figures S7 and S8). Similarly, povidone iodine also showed conclusive evidence of a significant effect (Final OR 0.3) (Electronic Supplementary Figure S9). In contrast, the evidence for Chlorhexidine combined with toothbrushing was deemed inconclusive, as its Z-curve did not cross the monitoring boundary, and the current sample size of 858 patients represents only 23.6% of its RIS of 3639 patients, indicating a clear need for further trials to reach a definitive conclusion for this specific intervention.

Since no significant differences were observed for other outcomes, neither cumulative meta-analysis nor trial sequential analysis were carried out.

### 3.10. Propensity Scores Matching Analysis

**Figure 11:**
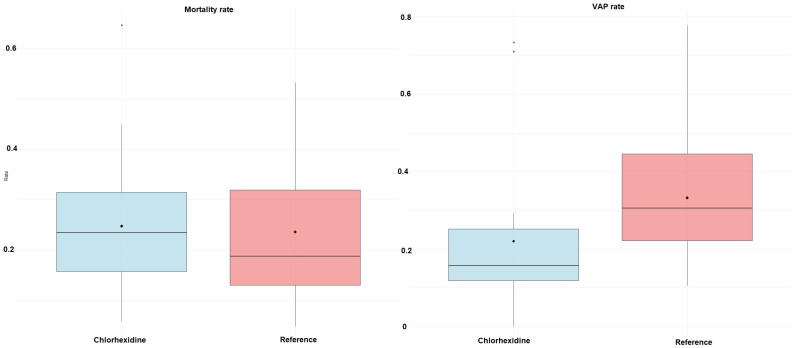
Box plots of propensity matched reduction in VAP and mortality with chlorhexidine.

The propensity scores matching analysis included 50 study arms comparing chlorhexidine-based oral care (n = 30) against reference interventions (n = 20). Optimal propensity score matching successfully created 20 matched pairs, achieving adequate covariate balance across all pre-specified confounders (all standardized mean differences <0.2). Chlorhexidine interventions demonstrated significantly lower VAP rates compared to reference interventions, with a mean VAP rate of 21.2% (SD = 17.9%) versus 33.3% (SD = 20.3%), respectively (Figure 11). This translated into an absolute risk reduction of 12.1 percentage points (95% CI: 1.8 to 22.2; *p* = 0.02) and a relative risk reduction of 36.3%. In sensitivity analyses, regression adjustment on the matched sample yielded consistent results, with chlorhexidine remaining significantly associated with reduced VAP rates (adjusted coefficient: −0.125, *p* = 0.033). No significant difference in mortality was observed between groups (mean difference: 1.6%, 95% CI: −10.2 to 7.1; *p* = 0.72). The consistency of findings across multiple matching approaches supports the robustness of the primary result, indicating that chlorhexidine-based oral care provides clinically important and statistically significant protection against VAP development in critically ill patients.

### 3.11. Grading the Strength of Evidence

Grades of the strength of evidence for key comparisons are summarized in Table 2. Moderate quality was observed for Chlorhexidine while very low strength was observed for other comparisons.

## 4. Discussion

### 4.1. Key Findings

Our network meta-analysis of 96 studies, encompassing over 20,000 critically ill patients, provides a comprehensive hierarchy of oropharyngeal interventions for VAP prevention. The central finding is that while numerous interventions, including antimicrobial combinations, chlorhexidine, povidone-iodine, and several herbal and combination strategies, demonstrate significant efficacy in reducing VAP incidence compared to standard care, no single intervention was consistently superior across all patient-centered outcomes. Interestingly, the reduction in VAP did not uniformly translate into improvements in mortality or resource utilization; only the combination of chlorhexidine with toothbrushing was associated with a significant reduction in mortality, and only antimicrobial combinations significantly reduced the duration of mechanical ventilation and ICU stay. This outcome-specific efficacy, confirmed by robust sensitivity and trial sequential analyses, underscores a critical disconnect between preventing VAP and achieving broader clinical benefits, suggesting that optimal intervention may depend on the specific therapeutic goal.

### 4.2. Comparison with Existing Literature

Our multivariate network meta-analysis elucidates a critical paradigm in VAP prevention: the efficacy of oropharyngeal interventions is profoundly outcome specific. We identified Chlorhexidine, Antimicrobial combinations, and Povidone iodine as the most effective agents for reducing VAP incidence. However, a pivotal finding was that only the combination of Chlorhexidine with toothbrushing was associated with a significant reduction in all-cause mortality. This suggests that the benefits of oral care extend beyond mere infection control and may be linked to the systemic modulation of inflammation and bacterial load through mechanical cleansing.

The role of chlorhexidine as a mainstay for VAP prevention is reinforced by our findings, which align with extensive prior evidence. For instance, a meta-analysis of 11 trials concluded that chlorhexidine significantly reduces VAP risk without impacting mortality [118]. This was further corroborated by the comprehensive Cochrane review of 40 trials, which positioned Chlorhexidine as a moderately effective yet reliable strategy [13]. Our analysis confirms this role but, through its network structure, reveals that while chlorhexidine is not the single most potent agent, it remains one of the most consistently effective and widely studied interventions across diverse clinical settings.

A central and novel insight from our study is the significant mortality benefit observed with the combination of Chlorhexidine and toothbrushing. While previous meta-analyses have often combined toothbrushing with various antiseptics without disaggregating their effects [13], our analysis was able to isolate this combination as uniquely beneficial for survival. The biological plausibility for this is strong. Toothbrushing effectively disrupts and removes dental plaque, a complex biofilm that serves as a reservoir for pathogens and a trigger for systemic inflammation [119]. The mechanical action of brushing is crucial for dislodging biofilm from protected niches, an effect that topical antiseptics alone cannot achieve. This is supported by in vitro evidence demonstrating that a combined approach of brushing and mouth rinse reduces biofilm thickness and bacterial load far more effectively than either intervention in isolation [120]. Therefore, the synergy of mechanical debridement and chemical antisepsis may be necessary to mitigate the systemic inflammatory burden that ultimately influences mortality risk.

Our results also highlight the superior efficacy of SOD with antimicrobial drug combinations (such as polymyxins, aminoglycosides, and antifungals) for reducing VAP incidence, duration of mechanical ventilation, and ICU length of stay. These regimens offer the advantage of targeted pathogen eradication, often through synergistic mechanisms, for example, polymyxins can disrupt the outer membrane of Gram-negative bacteria, enhancing the penetration and efficacy of aminoglycosides [121]. However, this potent efficacy is counterbalanced by the formidable threat of promoting antimicrobial resistance [122,123]. As these same antibiotic classes are last-line defenses for treating multidrug-resistant infections in the ICU, the widespread, prophylactic use of antimicrobial drugs necessitates careful consideration within institutional antimicrobial stewardship programs.

Beyond conventional agents, our analysis identified several herbal products, including Chamomile, Miswak, and Propolis, with point estimates suggesting superior VAP prevention compared to Chlorhexidine. This finding is consistent with a previous meta-analysis that reported a significant protective effect for miswak [12]. While the current evidence for these natural compounds is limited by smaller sample sizes and a higher risk of bias, they represent a promising and potentially cost-effective area for future research, particularly in resource-limited settings or as alternatives in the face of emerging antiseptic resistance.

Based on the findings of this analysis, we propose a paradigm shift towards a more nuanced and outcome-driven approach to oropharyngeal care in the ICU. First, clinical practice guidelines and institutional VAP prevention bundles should be updated to mandatorily incorporate toothbrushing as a foundational component, with the combination of Chlorhexidine and toothbrushing strongly considered as a first-line strategy given its unique association with reduced mortality. Second, the use of potent topical antimicrobial combinations, while highly effective for VAP prevention and resource use, must be carefully balanced against the critical imperative of antimicrobial stewardship; their implementation should be reserved for high-risk settings with robust resistance surveillance. Finally, the research agenda must evolve to address the identified evidence gaps. Future trials should be powered for patient-centered outcomes like mortality and should directly compare the most promising strategies from this network meta-analysis, such as chlorhexidine-toothbrushing versus a leading antimicrobial combination, to definitively guide clinical practice.

### 4.3. Strengths and Limitations

This network meta-analysis possesses several strengths, including its comprehensive and systematic assessment of a wide spectrum of pharmacological and herbal interventions on a unified platform, its adherence to rigorous PRISMA guidelines, and the use of advanced statistical methods like TSA and PSM analyses to confirm the robustness of key findings. However, several limitations warrant consideration. The overall certainty of evidence was predominantly low to very low, as per GRADE assessment, due to heterogeneity in VAP definitions, intervention protocols, and baseline care standards (VAP bundle adequacy) and limited studies due to which risk of bias could not be assessed, except for chlorhexidine. The heavy reliance on chlorhexidine as a common comparator, while creating a well-connected network, also limited the precision of estimates for many promising but less-studied interventions. Furthermore, the aggregation of varied antimicrobial combinations into a single node, while necessary for analysis, may obscure the efficacy of specific antibiotic regimens. Also, moderate to substantial statistical heterogeneity was observed for some outcomes. For clinicians, these results underscore that the selection of an oral care strategy should be a deliberate decision based on the primary goal, whether it is VAP prevention, mortality reduction, or minimizing resource use, as no single agent excels in all domains. For researchers, these findings highlight critical evidence gaps. Future studies should prioritize direct head-to-head comparisons of the most promising agents (Antimicrobial combinations versus Chlorhexidine and Chlorhexidine + Toothbrush), standardize VAP diagnostic criteria and oral care protocols, and are powered to detect differences in patient-centered outcomes like mortality and ICU length of stay, rather than solely on VAP incidence.

## 5. Conclusions

In conclusion, this comprehensive network meta-analysis establishes that effective VAP prevention is not monolithic but intervention specific. While antimicrobial combinations, Chlorhexidine, and Povidone iodine significantly reduce the incidence of VAP, their benefits do not uniformly extend to mortality or resource utilization. The combination of chlorhexidine with toothbrushing emerges as a critical strategy for reducing mortality, whereas antimicrobial combinations show unique promise in shortening the duration of mechanical ventilation and ICU stay. Consequently, the optimal oropharyngeal care strategy in the ICU should be tailored, moving beyond a one-size-fits-all approach to one that is deliberately aligned with specific patient outcomes and institutional priorities. These findings provide a crucial evidence base to refine VAP prevention protocols and guide future clinical research.

## Figures and Tables

**Table 1:** Key characteristics of included studies.

Study Id	Year	Country	ICU Setting	Study Design	VAP Bundle Adequacy	Interventions	Toothbrush Co-Intervention	Concentration (%)	Daily Frequency	Total Numbers	Mean Age (Years)	Male (%)	Severity of Illness Scale	Severity Score	VAP Definition
Abele-Horn [22]	1997	Germany	Anesthesiology	RCT	1	Antimicrobial combination	No	2	4	58	39.9	50	APACHE II	16	CPIS
						Standard of care	No	NA	Not mentioned	30	44.7	80	APACHE II	18	CPIS
Bellissimo-Rodrigues [23]	2014	Brazil	Mixed	RCT	0	Chlorhexidine + Toothbrush	Yes	2 or 0.12	Not mentioned	NA	NA	NA	Not provided	NA	NA
						Chlorhexidine	No	2 or 0.12	Not mentioned	NA	NA	NA	Not provided	NA	NA
Bergmans [24]	2001	Netherlands	Mixed	RCT	2	Antimicrobial combination	No	2	4	87	56.6	68	APACHE II	21	CDC
						Water	No	NA	4	61	58.7	77	APACHE II	21.2	CDC
Berry [25]	2011	Australia	Mixed	RCT	1	Water + Toothbrush	Yes	NA	12	78	59.1	56.4	APACHE II	21.64	Pugin’s
						Sodium bicarbonate + Toothbrush	Yes	NA	12	76	60.4	55.3	APACHE II	22	Pugin’s
						Chlorhexidine + Toothbrush	Yes	0.2	12	71	58.2	49.3	APACHE II	22.8	Pugin’s
Berry [26]	2013	Australia	Mixed	RCT	4	Listerine + Toothbrush	Yes	NA	2	127	59.96	57.5	APACHE II	21.21	Pugin’s
						Sodium bicarbonate + Toothbrush	Yes	NA	12	133	54.93	59.4	APACHE II	21.38	Pugin’s
						Water + Toothbrush	Yes	NA	12	138	58.82	60.9	APACHE II	20.86	Pugin’s
Chacko [27]	2017	India	Medical	RCT	2	Chlorhexidine	No	0.2	8	104	45.91	67.3	Not provided	NA	CDC
						Chlorhexidine + Toothbrush	Yes	0.2	8	102	41.02	44.1	Not provided	NA	CDC
Chen [28]	2016	China	Emergency	RCT	2	Antimicrobial drug	No	0.08	2	40	64.7	60	APACHE II	16.1	CPIS
						Chlorhexidine	No	0.2	2	155	62.6	99	APACHE II	16.1	CPIS
Chua [29]	2004	Philippines	Mixed	RCT	2	Povidone iodine	No	1	3	22	51.4	31.8	APACHE II	15.9	CDC
						Water	No	NA	3	20	55.2	50	APACHE II	17	CDC
Dahiya [30]	2012	India	Medical	RCT	0	Chlorhexidine	No	0.2	2	35	NA	NA	Not provided	NA	Not specified
						Hydrogen peroxide	No	NA	2	35	NA	NA	Not provided	NA	Not specified
Darbanian [31]	2024	Iran	Medical	RCT	2	Propolis	No	0.06	2	55	NA	49.1	Not provided	NA	CPIS
						Chlorhexidine	No	0.2	2	55	NA	49.1	Not provided	NA	CPIS
de Lacerda Vidal [32]	2017	Brazil	Mixed	RCT	3	Chlorhexidine + Toothbrush	Yes	0.12	2	105	59.4	48.6	APACHE II	21.9	ATS/IDSA
						Chlorhexidine	No	0.12	2	108	63.2	50	APACHE II	22.2	ATS/IDSA
De Riso [33]	1996	USA	Cardiothoracic	RCT	1	Chlorhexidine	No	0.12	2	173	64.1	68.8	Not provided	NA	NA
						Water	No	NA	2	180	63.5	68.3	Not provided	NA	NA
Deschepper [34]	2018	Belgium	Mixed	Cohort	0	Chlorhexidine	No	0.05 or 0.12	2 or 3	NA	NA	NA	Not provided	NA	NA
						No Chlorhexidine	No	NA	2 or 3	NA	NA	NA	Not provided	NA	NA
Dobakhti [35]	2023	Iran	Surgical	RCT	0	Chlorhexidine	No	0.2	3	32	NA	62.5	APACHE II	18.12	CPIS
						Chlorhexidine	No	0.2	3	32	NA	56.2	APACHE II	18.9	CPIS
Dobakhti [36]	2023	Iran	Surgical	RCT	0	Rose water + Chlorhexidine	No	0.12	3	40	45.75	60	APACHE II	18.25	CPIS
						Chlorhexidine	No	0.12	3	40	45.99	62.5	APACHE II	19.37	CPIS
Enwere [37]	2016	USA	Surgical	Cohort	2	Chlorhexidine + Toothbrush	Yes	0.12	2	64	57	67.2	Not provided	NA	CDC
						Toothbrush	Yes	NA	2	94	59	71.3	Not provided	NA	CDC
Feng [38]	2012	China	Medical	RCT	0	Povidone iodine + Toothbrush	Yes	0.05	4	71	43.7	NA	Not provided	NA	CSRD
						Nitrofurazone + Toothbrush	Yes	NA	4	65	38.5	NA	Not provided	NA	CSRD
						Saline + Toothbrush	Yes	NA	4	68	40.3	NA	Not provided	NA	CSRD
Fourrier [39]	2000	France	Medical	RCT	0	Chlorhexidine	No	0.2	3	30	51.2	63.3	APACHE II	37	NA
						Sodium bicarbonate + Saline	No	NA	4	30	50.4	63.3	APACHE II	33	NA
Fourrier [40]	2005	France	Medical	RCT	0	Chlorhexidine	No	0.2	3	114	61	72.8	Not provided	NA	Clinical + Radiological + Microbiological
						Placebo	No	NA	3	114	61.1	64	Not provided	NA	Clinical + Radiological + Microbiological
Franata [41]	2025	Indonesia	Unclear	RCT	0	Chlorhexidine	No	0.12	2	10	39.2	NA	Not provided	NA	CPIS
						Fluoride toothpaste + Toothbrush	Yes	NA	2	10	48.5	NA	Not provided	NA	CPIS
Galhardo [42]	2020	Brazil	Unclear	Cohort	0	Chlorhexidine + Toothbrush	Yes	0.12	Not mentioned	329	NA	NA	Not provided	NA	Clinical
						No Chlorhexidine	No	NA	Not mentioned	229	NA	NA	Not provided	NA	Clinical
Genuit [43]	2000	USA	Surgical	Non-randomized	0	Chlorhexidine	No	0.12	2	56	68.8	100	Not provided	NA	NA
						No Chlorhexidine	No	NA	Not mentioned	39	69.2	98	Not provided	NA	NA
Gharebaghi [44]	2020	Iran	Medical	RCT	0	Antimicrobial combination	No	2	4	52	NA	NA	Not provided	NA	Clinical + Radiological + Microbiological
						Chlorhexidine	No	0.2	4	53	NA	NA	Not provided	NA	Clinical + Radiological + Microbiological
Gholami [45]	2021	Iran	Unclear	RCT	0	*Zataria multiflora Boiss*.	No	10	3	30	NA	73.3	Not provided	NA	NA
						Chlorhexidine	No	0.2	3	30	NA	66.7	Not provided	NA	NA
						Saline	No	NA	3	30	NA	46.7	Not provided	NA	NA
Haghighat [46]	2022	Iran	Mixed	RCT	0	Chlorhexidine	No	0.2	2	20	NA	70	Not provided	NA	CPIS
						Chlorhexidine + Toothbrush	Yes	0.2	2	40	NA	62.5	Not provided	NA	CPIS
Hanifi [47]	2017	Iran	Surgical	RCT	0	Ozonated water	No	NA	3	39	44.42	64.1	Not provided	NA	CPIS
						Chlorhexidine	No	0.2	3	30	44.61	71.4	Not provided	NA	CPIS
Hashemi [48]	2018	Iran	Unclear	RCT	0	Chlorhexidine	No	0.2	4	43	48.44	60.46	Not provided	NA	CDC
						Stop-snoring herbal mouthwash	No	NA	4	43	54.06	60	Not provided	NA	CDC
Hu [49]	2009	China	Unclear	RCT	0	Saline	No	NA	2	25	NA	64	Not provided	NA	Clinical + Radiological + Microbiological
						Saline	No	NA	2	22	NA	59.1	Not provided	NA	Clinical + Radiological + Microbiological
Long [50]	2012	China	Unclear	RCT	0	Povidone iodine + Toothbrush	Yes	NA	3	31	60.06	64.5	APACHE II	17.94	Clinical + Radiological + Microbiological
						Povidone iodine	No	NA	3	30	63.67	60	APACHE II	18.23	Clinical + Radiological + Microbiological
Mo [51]	2016	China	Cardiothoracic	RCT	0	Saline	No	NA	4	105	59.14	57.1	Not provided	NA	CSRD
						Saline	No	NA	4	105	56.71	64.8	Not provided	NA	CSRD
Xu [52]	2007	China	Unclear	RCT	0	Saline	No	NA	2	44	NA	NA	Not provided	NA	CSRD
						Saline	No	NA	2	58	NA	NA	Not provided	NA	CSRD
						Saline	No	NA	2	62	NA	NA	Not provided	NA	CSRD
Xu [53]	2008	China	Unclear	RCT	0	Saline	No	NA	2	64	NA	NA	Not provided	NA	CSRD
						Saline	No	NA	2	52	NA	NA	Not provided	NA	CSRD
Zhao [54]	2012	China	Unclear	RCT	0	Triclosan	No	NA	4	162	NA	NA	Not provided	NA	Microbiological
						Saline	No	NA	4	162	NA	NA	Not provided	NA	Microbiological
Irani [55]	2019	Iran	Surgical	RCT	1	Miswak + Toothbrush	Yes	NA	2	35	33.65	82.9	Not provided	NA	CPIS
						Chlorhexidine	No	0.2	2	35	34.83	74.3	Not provided	NA	CPIS
Izadi [56]	2023	Iran	Unclear	RCT	0	Chlorhexidine	No	0.2	3	37	63.7	34.2	Not provided	NA	CPIS
						Ozonated water	No	NA	3	36	60.3	58.3	Not provided	NA	CPIS
Jacomo [57]	2011	Brazil	Cardiothoracic	RCT	0	Chlorhexidine	No	0.12	2	87	1.02	48	PRISM	3	CDC
						Water	No	NA	2	73	0.9	48	PRISM	3	CDC
Jahanshir [58]	2023	Iran	Unclear	RCT	0	Clove	No	6.66	2	84	55.21	61.9	Not provided	NA	CPIS
						Chlorhexidine	No	0.12	2	84	59.7	67.9	Not provided	NA	CPIS
Jamshidi [59]	2015	Iran	Unclear	RCT	0	Chlorhexidine	No	0.2	3	60	41.3	53.9	Not provided	NA	CPIS
						Toothbrush	Yes	NA	3	61	41.3	53.9	Not provided	NA	CPIS
						Chlorhexidine + Toothbrush	Yes	0.2	3	59	41.3	53.9	Not provided	NA	CPIS
Karakaya [60]	2022	Turkey	PICU	RCT	3	Chlorhexidine	No	0.12	6	88	4.3	47.7	PRISM	18	CDC
						Saline	No	0.9	6	86	3.2	30.2	PRISM	18	CDC
Kawyannejad [61]	2020	Iran	Unclear	RCT	3	Satureja plant	No	NA	3	40	42.22	57.5	Not provided	NA	CPIS
						Chlorhexidine	No	0.2	3	40	44.76	60	Not provided	NA	CPIS
Khaky [62]	2018	Iran	Unclear	RCT	0	Hydrogen peroxide + Silver ions	No	NA	3	37	41.6	72.5	SOFA	7.5	CPIS
						Chlorhexidine	No	2	3	38	44.1	67.5	SOFA	7.3	CPIS
Kiabi [63]	2023	Iran	Unclear	RCT	2	Persica	No	NA	2	25	NA	45.04	SOFA	8.8	CPIS
						Chlorhexidine	No	0.2	2	25	NA	45.04	SOFA	7.72	CPIS
Klarin [64]	2018	Sweden	Medical	RCT	0	Probiotic	No	NA	2	69	66	58	APACHE II	22	Clinical + Radiological + Microbiological
						Chlorhexidine + Toothbrush	Yes	0.1	2	68	65.5	52.9	APACHE II	24	Clinical + Radiological + Microbiological
Koeman [65]	2006	Netherlands	Mixed	RCT	2	Chlorhexidine	No	2	4	127	60.9	52	APACHE II	22.2	Clinical + Radiological + Microbiological
						Chlorhexidine + Colistin	No	2	4	128	62.4	56	APACHE II	23.7	Clinical + Radiological + Microbiological
						Saline	No	0.9	4	130	62.1	72	APACHE II	21.8	Clinical + Radiological + Microbiological
Kollef [66]	2006	Multinational	Mixed	RCT	0	Iseganan	No	NA	6	362	60.5	62.9	APACHE II	19.6	Clinical + Radiological + Microbiological
						Placebo	No	NA	6	347	57.5	57.5	APACHE II	19.3	Clinical + Radiological + Microbiological
Kushara [67]	2012	Brazil	PICU	RCT	2	Chlorhexidine + Toothbrush	Yes	0.12	2	46	1	60.9	Not provided	NA	CPIS/CDC/NHSN
						Toothbrush	Yes	NA	2	50	2.9	64	Not provided	NA	CPIS/CDC/NHSN
Lev [68]	2015	Israel	Mixed	RCT	0	Sodium bicarbonate + Hydrogen peroxide + Vitamin E + Toothbrush	Yes	NA	3	45	68.7	55.5	APACHE II	19.1	NHSN
						Chlorhexidine	No	0.2	3	45	71.8	53.3	APACHE II	18.2	NHSN
Leyderman [69]	2024	Russia	Surgical	RCT	3	Chlorhexidine + Toothbrush	Yes	0.05	3	25	65	NA	APACHE II	13.05	CPIS
						Chlorhexidine	No	0.05	2	22	70	NA	APACHE II	13.1	CPIS
Li [70]	2021	China	NICU	RCT	0	Colostrum + Sodium bicarbonate	No	2.5	4	40	NA	NA	Not provided	NA	Clinical + Radiological + Microbiological
						Colostrum	No	NA	4	40	NA	NA	Not provided	NA	Clinical + Radiological + Microbiological
						Sodium bicarbonate	No	2.5	4	40	NA	NA	Not provided	NA	Clinical + Radiological + Microbiological
Li-Yin [71]	2011	Taiwan	Surgical	RCT	2	Water + Toothbrush	Yes	NA	2	28	60.7	60.7	APACHE II	19.6	CPIS
						Water	No	NA	2	25	60.5	68	APACHE II	19.4	CPIS
Lorente [72]	2012	Spain	Mixed	RCT	3	Chlorhexidine + Toothbrush	Yes	0.12	3	217	61	67.3	APACHE II	17.88	Clinical + Radiological + Microbiological
						Chlorhexidine	No	0.12	3	219	60.4	66.2	APACHE II	19.16	Clinical + Radiological + Microbiological
Maarefvand [73]	2015	Iran	Unclear	RCT	0	Chamomile	No	NA	2	30	45.93	46.7	APACHE II	17.7	CPIS
						Chlorhexidine	No	0.12	2	60	51.63	50	APACHE II	17.63	CPIS
Meidani [74]	2018	Iran	Unclear	RCT	0	Chlorhexidine	No	0.2	3	50	50.6	74	Not provided	NA	CDC
						Potassium permanganate	No	0.01	3	50	49.8	74	Not provided	NA	CDC
						Placebo	No	NA	3	50	51.7	66	Not provided	NA	CDC
Meinberg [75]	2012	Brazil	Surgical	RCT	0	Chlorhexidine + Toothbrush	Yes	2	4	28	40.1	NA	APACHE II	17.9	Clinical + Radiological + Microbiological
						Toothbrush	Yes	2	4	24	41	NA	APACHE II	16.7	Clinical + Radiological + Microbiological
Moghaddam [76]	2022	Iran	Unclear	RCT	2	Chamomile + Toothbrush	Yes	10	2	40	45.1	NA	Not provided	NA	CPIS
						Chlorhexidine + Toothbrush	Yes	0.2	2	40	39.85	NA	Not provided	NA	CPIS
Mohseni [77]	2024	Iran	Unclear	RCT	2	Tea tree oil + Aloe vera+ Toothbrush	Yes	NA	2	31	45	64.5	Not provided	NA	CPIS
						Chlorhexidine + Toothbrush	Yes	0.2	2	31	45	64.5	Not provided	NA	CPIS
Mori [78]	2006	Japan	Mixed	Cohort	0	Povidone iodine	No	Not known	8	1248	53	62	APACHE II	13.9	Clinical + Radiological + Microbiological
						No oral care	No	NA	NA	414	53	66	APACHE II	13.4	Clinical + Radiological + Microbiological
Nasiriani [79]	2016	Iran	Unclear	RCT	0	Chlorhexidine + Saline+ Toothbrush	Yes	Not known	2	84	44.9	66.7	Not provided	NA	CPIS
						Chlorhexidine + Saline	No	Not known	2	84	44.2	67.9	Not provided	NA	CPIS
Nicolosi [80]	2014	Argentina	Cardiothoracic	Cohort	0	Chlorhexidine + Toothbrush	Yes	0.12	2	150	62.3	81.3	Not provided	NA	Clinical + Radiological
						Standard of care	No	NA	2	150	63.1	86	Not provided	NA	Clinical + Radiological
Nobahar [81]	2016	Iran	Mixed	RCT	1	Hydrogen peroxide	No	3	2	34	66	50	Not provided	NA	CPIS
						Saline	No	0.9	2	34	63.4	47.1	Not provided	NA	CPIS
Ory [82]	2017	France	Mixed	Cohort	0	Chlorhexidine	No	0.5	3	932	64.6	69.3	SAPS2	54	Clinical + Radiological + Microbiological
						Chlorhexidine + Toothbrush	Yes	0.5	3	1151	63.6	67.5	SAPS2	53.3	Clinical + Radiological + Microbiological
Ozcaka [83]	2012	Turkey	Respiratory	RCT	0	Chlorhexidine	No	0.2	4	29	60.5	NA	APACHE II	23.9	Not specified
						Saline	No	0.9	4	32	56	NA	APACHE II	24.7	Not specified
Panchabai [84]	2009	India	Medical	RCT	3	Chlorhexidine	No	0.2	2	88	NA	NA	Not provided	NA	Clinical + Radiological + Microbiological
						Potassium permanganate	No	0.01	2	83	NA	NA	Not provided	NA	Clinical + Radiological + Microbiological
Pedreira [85]	2009	Brazil	PICU	RCT	0	Chlorhexidine + Toothbrush	Yes	0.12	2	27	NA	NA	Not provided	NA	NA
						Toothbrush	No	NA	2	29	NA	NA	Not provided	NA	NA
Pobo [86]	2009	Spain	Mixed	RCT	1	Chlorhexidine	No	0.12	3	73	52.6	63	APACHE II	18.7	ATS/IDSA
						Chlorhexidine + Toothbrush	Yes	0.12	3	74	55.3	66.2	APACHE II	18.8	ATS/IDSA
Pugin [87]	1991	Switzerland	Surgical	RCT	1	Antimicrobial combination	No	NA	1	25	45	76	APACHE II	15.8	CPIS
						Placebo	No	NA	1	27	46	74.1	APACHE II	14.7	CPIS
Rezvani [88]	2018	Iran	Mixed	RCT	0	Chamomile	No	NA	3	45	60.78	60	Not provided	NA	Not specified
						Chlorhexidine	No	0.2	3	45	59.18	55	Not provided	NA	Not specified
Rodriguez-Roldan [89]	1990	Spain	Mixed	RCT	1	Chlorhexidine + Antimicrobial combination	N0	0.1	4	13	54	53.8	APACHE II	16	Clinical + Radiological + Microbiological
						Chlorhexidine	No	0.1	4	15	49	66.7	APACHE II	18	Clinical + Radiological + Microbiological
Kes [90]	2021	Turkey	Surgical	RCT	0	Chlorhexidine	No	0.12	3	29	72.79	62.1	APACHE II	16.1	CPIS
						Sodium bicarbonate	No	NA	3	28	77.37	57.1	APACHE II	16.6	CPIS
Houston [91]	2002	USA	Cardiothoracic	RCT	0	Chlorhexidine	No	0.12	2	19	NA	NA	Not provided	NA	CDC
						Listerine	No	NA	2	18	NA	NA	Not provided	NA	CDC
Saito [92]	2022	Vietnam	Mixed	Cohort	1	Toothbrush	Yes	NA	Not mentioned	300	57.5	56.3	APACHE II	20	CPIS
						Standard of care	No	NA	Not mentioned	303	56	58.1	APACHE II	17	CPIS
Scannapieco [93]	2009	USA	Unclear	RCT	0	Chlorhexidine	No	0.12	1	47	44.8	91.5	APACHE II	18.5	CPIS
						Chlorhexidine	No	0.12	2	50	47.6	88	APACHE II	19.7	CPIS
						Water	No	0.12	1	49	50	73.5	APACHE II	19.1	CPIS
Sebastian [94]	2012	India	PICU	RCT	2	Chlorhexidine	No	1	3	41	NA	56.1	PIM2	19	CDC
						Placebo	No	NA	3	45	NA	60	PIM2	23.5	CDC
Seguin [95]	2006	France	Surgical	RCT	2	Povidone iodine	No	10	6	36	38	78	SAPS2	39	BAL + Microbiological
						Saline	No	0.9	6	31	38	77	SAPS2	41	BAL + Microbiological
						Standard of care	No	NA	6	31	41	74	SAPS2	40	BAL + Microbiological
Seguin [96]	2013	France	Surgical	RCT	2	Povidone iodine	No	10	6	85	48	71	SAPS2	47	Clinical + Radiological + Microbiological
						Water	No	NA	6	82	48	78	SAPS2	46	Clinical + Radiological + Microbiological
Sharma [97]	2012	India	Mixed	RCT	0	Chlorhexidine	No	0.12	2	130	NA	74.6	Not provided	NA	CPIS
						Saline	No	0.9	2	130	NA	72.3	Not provided	NA	CPIS
Shorofi [98]	2025	Iran	Mixed	RCT	0	*Zataria multiflora Boiss.* + Chlorhexidine	No	0.2	2	60	46.57	66.7	Not provided	NA	CPIS
						Chlorhexidine	No	0.2	2	60	50.58	66.7	Not provided	NA	CPIS
Singh [99]	2022	India	Unclear	RCT	3	Chlorhexidine + Toothbrush	Yes	0.2	2	110	39.02	50	Not provided	NA	Not specified
						Chlorhexidine	No	0.2	2	110	39.1	50.9	Not provided	NA	Not specified
Siriyanyongwong [100]	2022	Thailand	Medical	RCT	0	Chlorhexidine + Moraceae + Toothbrush	Yes	0.02	3	15	68	53.3	SOFA	9	CDC
						Chlorhexidine	No	0.12	3	15	63	53.3	SOFA	8	CDC
Stefanescu [101]	2013	USA	NICU	RCT	1	Biotene	No	NA	6	20	0.5	35	APGAR	3	CDC
						Water	No	NA	6	21	0.48	52	APGAR	4	CDC
Takeyasu [102]	2014	Japan	Unclear	RCT	2	Povidone iodine + Toothbrush	Yes	1	3	84	67.9	65.5	Not provided	NA	Not specified
						Toothbrush	Yes	NA	3	58	67.9	65.5	Not provided	NA	Not specified
Tantipong [103]	2008	Thailand	Mixed	RCT	0	Chlorhexidine + Toothbrush	Yes	2	4	102	56.5	49.1	APACHE II	16.7	Clinical + Radiological + Microbiological
						Saline + Toothbrush	Yes	0.9	4	105	60.3	48.6	APACHE II	18.2	Clinical + Radiological + Microbiological
Tuon [104]	2017	Brazil	Mixed	RCT	0	Chlorhexidine	No	2	2	8	53.1	62.5	APACHE II	NA	CDC
						Saline	No	0.9	2	8	42.8	50	Not provided	NA	CDC
Vyas [105]	2020	India	Unclear	RCT	1	Chlorhexidine	No	0.12	3	70	47	78.57	Not provided	NA	CPIS
						Chlorhexidine	No	0.2	3	70	48.5	68.57	Not provided	NA	CPIS
Yadav [106]	2022	India	Unclear	RCT	1	Antimicrobial drug	No	2	4	82	33.21	50	APACHE II	21.9	CPIS
						Chlorhexidine	No	2	4	69	33.75	47.8	APACHE II	22.58	CPIS
Yu [107] & [108]	2021	China	NICU	RCT	0	Breast milk	No	NA	8	31	0.15	NA	Not provided	NA	Clinical + Radiological + Microbiological
						Saline	No	NA	8	31	0.14	NA	Not provided	NA	Clinical + Radiological + Microbiological
						Sodium bicarbonate	No	NA	8	31	0.15	NA	Not provided	NA	Clinical + Radiological + Microbiological
Zand [109]	2017	Iran	Mixed	RCT	1	Chlorhexidine	No	0.2	2	57	45.43	80.7	APACHE IV	61.33	CPIS
						Chlorhexidine	No	2	2	57	44.45	80.7	APACHE IV	56.01	CPIS
Zarinfar [110]	2021	Iran	Mixed	RCT	4	Standard of care	No	NA	Not mentioned	43	60.4	48.8	APACHE II	NA	CPIS
						Chlorhexidine	No	0.12	2	43	53.5	60.5	APACHE II	NA	CPIS
						Chlorhexidine	No	0.12	2	43	50	44.2	APACHE II	NA	CPIS
Bellissimo-Rodrigues [111]	2009	Brazil	Mixed	RCT	2	Chlorhexidine	No	0.12	3	98	62.5	48	APACHE II	17	CDC
						Placebo	No	NA	3	96	54	53	APACHE II	19	CDC
Bopp [112]	2006	USA	Mixed	RCT	0	Chlorhexidine	No	0.12	2	2	40	50	Not provided	NA	Not specified
						Hydrogen peroxide or Listerine	No	NA	6	3	73.7	33	Not provided	NA	Not specified
Cabov [113]	2010	Croatia	Surgical	RCT	0	Chlorhexidine	No	0.2	3	30	57	63.3	SAPS2	30	Clinical + Radiological + Microbiological
						Placebo	No	NA	3	30	52	66.7	SAPS2	28.2	Clinical + Radiological + Microbiological
de Smet [114]	2009	Netherlands	Mixed	RCT	0	Antimicrobial combination	No	2	4	1904	61.4	63.7	APACHE II	19.5	Not specified
						Standard of care	No	NA	Not mentioned	1990	61.4	61.3	APACHE II	18.6	Not specified
Loha [115]	2022	India	Unclear	RCT	5	Chlorhexidine	No	2	3	44	56.86	50	APACHE II	21.21	CPIS
						Chlorhexidine + Sodium bicarbonate	No	2 & 0.9	2	44	54.96	47.7	APACHE II	21.28	CPIS
Munro [116]	2009	USA	Mixed	RCT	0	Toothbrush	Yes	NA	3	49	47.1	57	APACHE III	76.4	Not specified
						Chlorhexidine	No	0.12	2	44	46.1	59	APACHE III	80.4	Not specified
						Chlorhexidine + Toothbrush	Yes	0.12	3	48	47.3	58	APACHE III	76.2	Not specified
						Standard of care	No	NA	Not mentioned	51	46.8	73	APACHE III	76.2	Not specified
Zambrano [117]	2024	Brazil	Unclear	RCT	0	Chlorhexidine	No	0.12	2	45	63	55	Not provided	NA	Not specified
						Chlorhexidine + Toothbrush	Yes	0.12	2	45	65	69	Not provided	NA	Not specified

RCT: Randomized clinical trial; CPIS: Clinical Pulmonary Infection Score; NA: Not available/Not applicable; CDC: Centers for Disease Control and Prevention; APACHE: Acute Physiology And Chronic Health Evaluation; ATS/IDSA: American Thoracic Society/Infectious Disease Society of America; CSRD: Chinese Society for Respiratory Disease; and NHSN: National Healthcare Safety Network; PICU: Pediatric intensive care unit; SOFA: Sequential organ failure assessment; PRISM: Pediatric Risk of Mortality; PIM 2: Pediatric index of mortality 2; APGAR: Appearance, Pulse, Grimace, Activity, and Respiration; SAPS2: Simplified acute physiology score II; BAL: Bronchoalveolar lavage.

**Table 2:** Grading the strength of estimates of key comparisons.

Comparisons with Reference Interventions for the Risk of VAP	Comparative Risks of VAP per 1000 Patients with Reference Interventions (95% Confidence Intervals)		Effect Estimates (ORs) and the Quality of Evidence
	Assumed Risk ^1^	Corresponding Risk	
Random effect estimates from network meta-analysis			
Chlorhexidine	311	199 (151 to 258)	0.55 [0.39, 0.77]; Moderate ^2^
Antimicrobial combination		123 (63 to 224)	0.31 [0.15, 0.64]; Very low ^2,3^
Povidone iodine		150 (87 to 243)	0.39 [0.21, 0.71]; Very low ^2,3^
Chlorhexidine + Toothbrush		147 (102 to 205)	0.38 [0.25, 0.57]; Very low ^2,3^
Toothbrush alone		172 (112 to 258)	0.46 [0.28, 0.77]; Very low ^2,3^
Miswak + Toothbrush		18 (0 to 260)	0.04 [0, 0.78]; Very low ^2,3^
Chamomile + Toothbrush		18 (4 to 67)	0.04 [0.01, 0.16]; Very low ^2,3^
Hydrogen peroxide + Silver ions		22 (0 to 190)	0.05 [0, 0.52]; Very low ^2,3^
Hyrogen peroxide+ Vitamin E + Toothbrush		47 (9 to 193)	0.11 [0.02, 0.53]; Very low ^2,3^
Nitrofurazone + Toothbrush		51 (13 to 156)	0.12 [0.03, 0.41]; Very low ^2,3^
Ozonated water		63 (22 to 178)	0.15 [0.05, 0.48]; Very low ^2,3^
Clove		79 (26 to 235)	0.19 [0.06, 0.68]; Very low ^2,3^
Propolis		87 (26 to 260)	0.21 [0.06, 0.78]; Very low ^2,3^
Satureja plant		90 (22 to 280)	0.22 [0.05, 0.86]; Very low ^2,3^
Random effect estimates from pairwise comparisons			
Chlorhexidine	300	208 (143 to 289)	0.61 [0.39, 0.95]; Moderate ^2^

VAP: Ventilator-associated pneumonia. 1—Assumed risk was the median risk of VAP with reference interventions across the studies; 2—Downgraded one level due to heterogeneity of studies; 3—Downgraded two levels as publication bias could not be assessed/ruled out. Moderate: The true effect of an intervention is close to the estimate, but there is a possibility it could be different; Very low: We have very little confidence in the effect estimate.

## Data Availability

The datasets used and/or analyzed during the current study are available from the corresponding author on reasonable request.

## Supplementary Materials

The following supporting information can be downloaded at: https://www.mdpi.com/article/10.3390/jcm14228174/s1, Electronic Supplementary File S1. PRISMA 2020 Checklist; Electronic Supplementary Table S1. Search strategy; Electronic Supplementary Table S2. Leave-one-out sensitivity analysis for the risk of VAP; Electronic Supplementary Table S3. Leave-one-out sensitivity analysis for the risk of mortality; Electronic Supplementary Table S4. Leave-one-out sensitivity analysis for the duration of mechanical ventilation; Electronic Supplementary Table S5. Leave-one-out sensitivity analysis for the duration of ICU stay; Electronic Supplementary Figure S1. Histogram of bootstrap meta-analyses; Electronic Supplementary Figure S2. Forest plots for sub-group analyses; Electronic Supplementary Figure S3. Cumulative meta-analysis plot for the risk of VAP for chlorhexidine compared to reference interventions; Electronic Supplementary Figure S4. Cumulative meta-analysis plot for the risk of VAP for antimicrobial combination compared to reference interventions; Electronic Supplementary Figure S5. Cumulative meta-analysis plot for the risk of VAP for Chlorhexidine + Toothbrush compared to reference interventions; Electronic Supplementary Figure S6. Cumulative meta-analysis plot for the risk of VAP for povidone iodine compared to reference interventions; Electronic Supplementary Figure S7. Trial sequential analysis for the risk of VAP for chlorhexidine compared to reference interventions; Electronic Supplementary Figure S8. Trial sequential analysis for the risk of VAP for antimicrobial combination compared to reference interventions; Electronic Supplementary Figure S9. Trial sequential analysis for the risk of VAP for povidone iodine compared to reference interventions.
