# Supplementary material for: Oropharyngeal Interventions in Intubated Patients for Preventing Ventilator Associated Pneumonia: A Systematic Review and Multi-Variate Network Meta-Analysis Evaluating Pharmacological Agents
Source: J Clin Med. 2025 Nov 18;14(22):8174. doi: 10.3390/jcm14228174 (PMC12653545; doi:10.3390/jcm14228174)
Supplement: Supplementary file 1 [file jcm-14-08174-s001.zip › jcm-3983009-supplementary.pdf]

Electronic Supplementary Table S1. Search strategy.

Database	Search strategy
**PubMed**	((chlorhexidine[Title/Abstract] OR toothbrush[Title/Abstract] OR antiseptic[Title/Abstract] OR potassium permanganate[Title/Abstract] OR ozonated water[Title/Abstract] OR nanosil[Title/Abstract] OR miswak[Title/Abstract] OR povidone iodine[Title/Abstract] OR saline[Title/Abstract] OR bicarbonate[Title/Abstract] OR triclosan[Title/Abstract] OR listerine[Title/Abstract] OR furacilin[Title/Abstract] OR biotene[Title/Abstract] OR hydrogen peroxide[Title/Abstract] OR vaseline[Title/Abstract] OR oral foam[Title/Abstract] OR iseganan[Title/Abstract] OR amphotericin B[Title/Abstract] OR tobramycin[Title/Abstract] OR polymyxin[Title/Abstract] OR gentamicin[Title/Abstract] OR colistin[Title/Abstract] OR vancomycin[Title/Abstract] OR chinese herb[Title/Abstract] OR persica[Title/Abstract] OR matrica[Title/Abstract] OR achillea millefolium[Title/Abstract] OR mentha spicata[Title/Abstract] OR chamomile[Title/Abstract]) AND (ventilation[Title/Abstract] OR ventilator[Title/Abstract] OR ventilator associated pneumonia[Title/Abstract] OR VAP[Title/Abstract]))
**Cochrane CENTRAL**	(chlorhexidine OR toothbrush OR antiseptic OR potassium permanganate OR ozonated water OR nanosil OR miswak OR povidone iodine OR saline OR bicarbonate OR triclosan OR listerine OR furacilin OR biotene OR hydrogen peroxide OR vaseline OR oral foam OR iseganan OR amphotericin B OR tobramycin OR polymyxin OR gentamicin OR colistin OR vancomycin OR chinese herb OR persica OR matrica OR achillea millefolium OR mentha spicata OR chamomile) AND (ventilation OR ventilator OR ventilator associated pneumonia OR VAP)
**EMBASE**	(chlorhexidine OR toothbrush OR antiseptic OR 'potassium permanganate' OR 'ozonated water' OR nanosil OR miswak OR 'povidone iodine' OR saline OR bicarbonate OR triclosan OR listerine OR furacilin OR biotene OR 'hydrogen peroxide' OR vaseline OR 'oral foam' OR iseganan OR 'amphotericin B' OR tobramycin OR polymyxin OR gentamicin OR colistin OR vancomycin OR 'chinese herb' OR persica OR matrica OR 'achillea millefolium' OR 'mentha spicata' OR 'Matricaria chamomilla extract'):ti,ab,kw AND ((ventilation OR ventilator OR 'ventilator associated pneumonia' OR VAP):ti,ab,kw)

Electronic Supplementary Table S2. Leave-one-out sensitivity analysis for the risk of VAP.

Antimicrobial combination

Study Removed	OR	CI_lower	CI_upper
Abele-Horn 1997 [22]	0.1565361	0.02300326	1.0652204
Bergmans 2001 [24]	0.1448214	0.02491062	0.8419396
Pugin 1991 [87]	0.3586646	0.18598131	0.6916838

Povidone iodine

Study_Removed	OR	CI_lower	CI_upper
Chua 2004 [29]	0.4319903	0.1425364	1.3092493
Mori 2006 [78]	0.5078526	0.1533684	1.6816654
Seguin 2006 [95]	0.6048047	0.2735045	1.3374137
Seguin 2013 [96]	0.3399315	0.2124470	0.5439165

Chlorhexidine

Study_Removed	OR	CI_lower	CI_upper
Fourrier 2005 [40]	0.5725325	0.3742315	0.8759111
Jacomo 2011 [57]	0.5645071	0.3725002	0.8554847
Karakaya 2022 [60]	0.5797998	0.3747643	0.8970115
Koeman 2006 [65]	0.6138911	0.3936348	0.9573908
Meidani 2018 [74]	0.6355030	0.4162553	0.9702315
Ozcaka 2012 [83]	0.6349587	0.4157785	0.9696811
Scannapieco 2009a [93]	0.6109915	0.3953469	0.9442608
Scannapieco 2009b [93]	0.6144276	0.3977830	0.9490635
Sebastian 2012 [94]	0.5842429	0.3793120	0.8998918
Sharma 2012 [97]	0.7223697	0.5556782	0.9390650

Tuon 2017 [104]	0.5774245	0.3854033	0.8651174
Zarinfar 2021a [110]	0.5893450	0.3809622	0.9117113
Zarinfar 2021b [110]	0.5950159	0.3838351	0.9223854

Electronic Supplementary Table S3. Leave-one-out sensitivity analysis for the risk of mortality.

Antimicrobial combination

Study_Removed	OR	CI_lower	CI_upper
Abele-Horn 1997 [22]	0.6443592	0.3536761	1.173952
Bergmans 2001 [24]	1.1418335	0.4910670	2.655002
Pugin 1991 [87]	0.6866179	0.3430821	1.374144

Povidone iodine

Study_Removed	OR	CI_lower	CI_upper
Chua 2004 [29]	0.8316038	0.2669982	2.590148
Seguin 2006 [95]	1.3192860	0.7254982	2.399063
Seguin 2013 [96]	0.6979560	0.2496075	1.951635

Chlorhexidine

Study_Removed	OR	CI_lower	CI_upper
De Riso 1996 [33]	0.9613474	0.7850627	1.177217
Deschepper 2018 [34]	0.9290788	0.6362315	1.356719
Fourrier 2005 [40]	0.8910287	0.7203338	1.102173
Genuit 2000 [43]	0.9465678	0.7718609	1.160819
Jacomo 2011 [57]	0.9385512	0.7658352	1.150219
Karakaya 2022 [60]	0.8997412	0.7320068	1.105911
Meidani 2018 [74]	0.9393340	0.7667504	1.150764
Scannapieco 2009a [93]	0.9317528	0.7594571	1.143137
Scannapieco 2009a [93]	0.9342494	0.7614957	1.146194
Sebastian 2012 [94]	0.9639332	0.7840359	1.185108

Electronic Supplementary Table S4. Leave-one-out sensitivity analysis for the duration of mechanical ventilation.

Povidone iodine

Study_Removed	MD	CI_lower	CI_upper
Chua 2004 [29]	-0.17841488	-1.170230	0.8134007
Mori 2006 [78]	-0.38193962	-2.815568	2.0516884
Seguin 2006 [95]	-0.06815363	-1.064041	0.9277342

Chlorhexidine

Study_Removed	MD	CI_lower	CI_upper
Fourrier 2005 [40]	0.4063881	0.037291878	0.7754843
Gholami 2021 [45]	0.5651897	-0.383311315	1.5136908
Jacomo 2011 [57]	0.3545151	-0.016857761	0.7258881
Karakaya 2022 [60]	0.4385568	-0.063927766	0.9410413
Koeman 2006 [65]	0.3859800	0.017805723	0.7541543
Meidani 2018 [74]	0.4268980	0.061242466	0.7925536
Ozaka 2012 [83]	0.4434430	0.078250549	0.8086354
Scannapieco 2009a [93]	0.4976861	-0.006725179	1.0020975
Scannapieco 2009b [93]	0.5400493	0.043446190	1.0366524
Sebastian 2012 [94]	0.4325765	0.066287268	0.7988658

Electronic Supplementary Table S5. Leave-one-out sensitivity analysis for the duration of ICU stay.

Povidone iodine

Study_Removed	MD	CI_lower	CI_upper
Chua 2004 [29]	0.2420334	-0.8224081	1.306475
Mori 2006 [78]	-0.3639332	-3.3039055	2.576039
Seguin 2006 [95]	0.1938095	-0.8641508	1.251770
Seguin 2013 [96]	0.2912323	-0.7833625	1.365827

Chlorhexidine

Study_Removed	MD	CI_lower	CI_upper
Fourrier 2005 [40]	1.1262407	-3.367776	5.620257
Gholami 2021 [45]	2.1137394	-1.607883	5.835362
Jacomo 2011 [57]	-0.6895745	-3.170416	1.791267
Karakaya 2022 [60]	0.8309768	-3.604747	5.266700
Koeman 2006 [65]	1.0386322	-3.407816	5.485080
Meidani 2018 [74]	1.0599641	-3.321664	5.441593
Ozcaka 2012 [83]	1.5565352	-2.639694	5.752764
Sebastian 2012 [94]	1.3916024	-2.996998	5.780203

Electronic Supplementary Figure S1. Histogram of bootstrap meta-analyses.

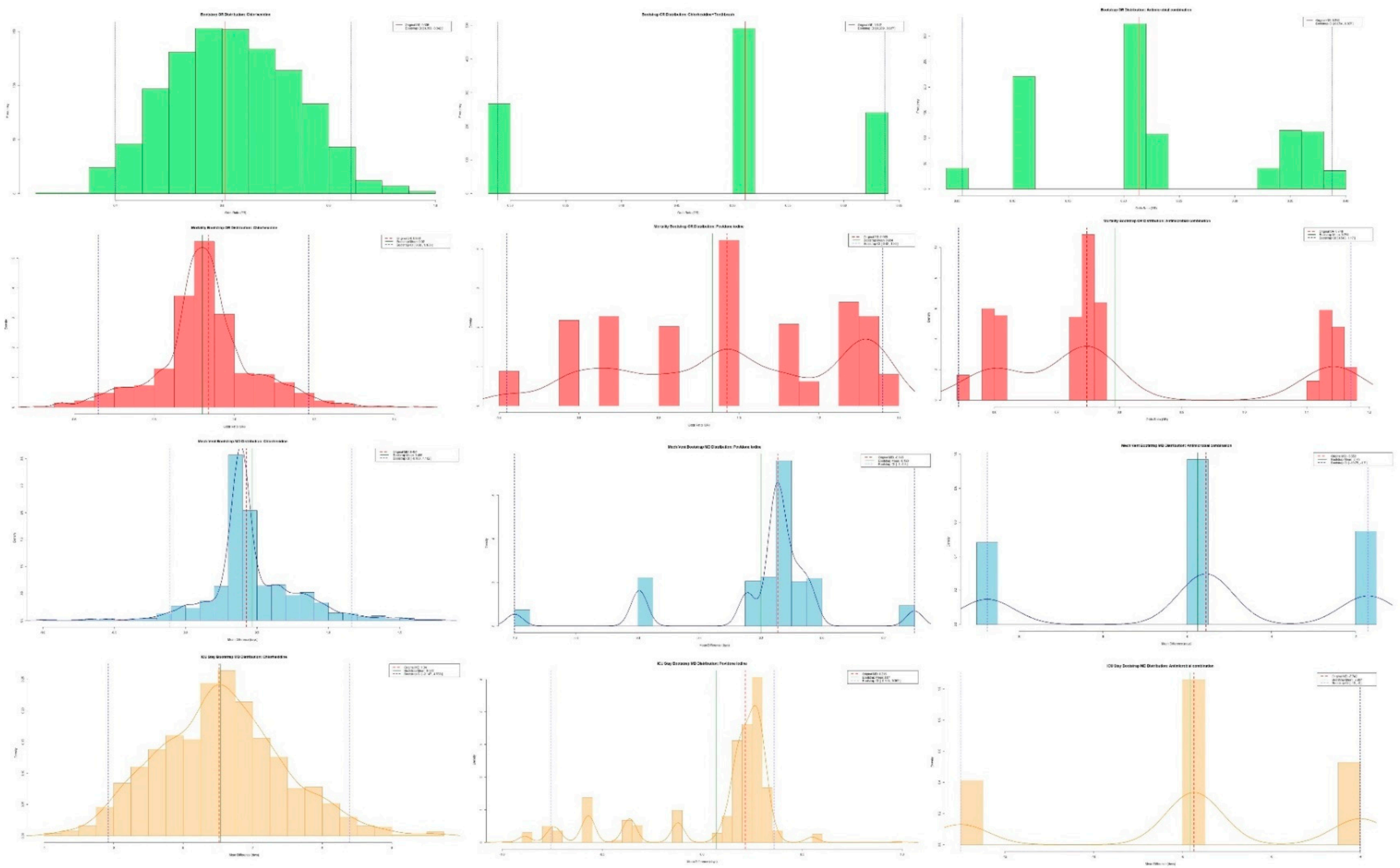

The different rows and colors likely correspond to different outcomes or sets of comparisons (the top row in green: VAP prevention; the second row in red: mortality; the third row in blue: duration of mechanical ventilation; and the bottom row in orange: ICU stay duration). The left-most column is related to chlorhexidine, middle column for povidone iodine, and the right-most column for antimicrobial combination.

Electronic Supplementary Figure S2. Forest plots for sub-group analyses.

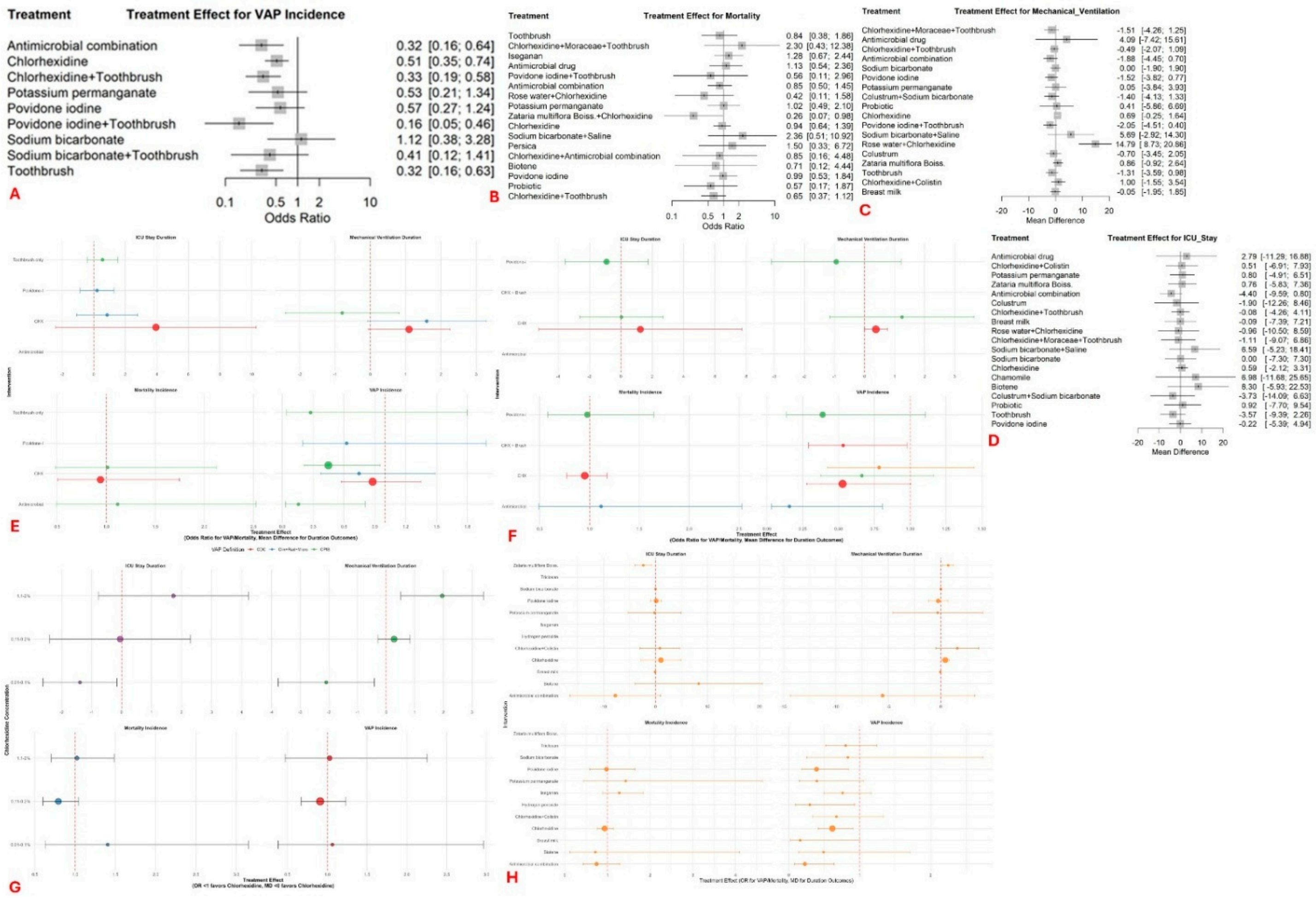

This figure depicts the pooled estimates from RCTs (A: for VAP; B: mortality; C: duration of mechanical ventilation; and D: duration of ICU stay); VAP definitions (E); adequacy of VAP bundle (F); chlorhexidine concentrations (G); and without toothbrushing as co-intervention (H). The vertical lines (either dashed or solid) are the lines of no difference. Circles and diamonds represent the pooled

effect estimates (odds ratios for VAP and mortality incidence; and mean difference for durations of mechanical ventilation and ICU stay).

Electronic Supplementary Figure S3. Cumulative meta-analysis plot for the risk of VAP for chlorhexidine compared to reference interventions.

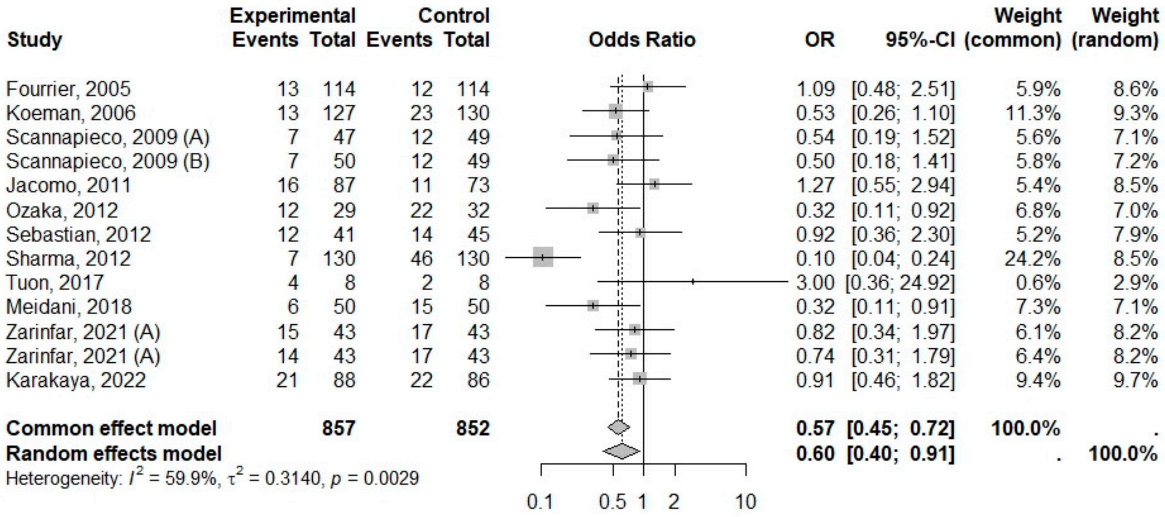

Fourrier 2005 [40], Koeman 2006 [65], Scannapieco 2009 A and B [93], Jacomo 2011 [57], Ozcaka 2012 [83], Sebastian 2012 [94], Sharma 2012 [97], Tuon 2017 [104], Meidani 2018 [74], Zarinfar 2021 A and B [110], Karakaya 2022 [60].

Electronic Supplementary Figure S4. Cumulative meta-analysis plot for the risk of VAP for antimicrobial combination compared to reference interventions.

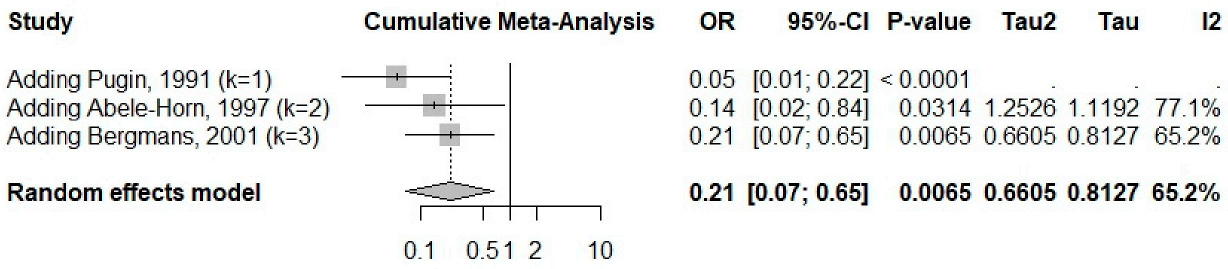

Pugin 1991 [87], Abele-Horn 1997 [22], Bergmans 2001 [24].

Electronic Supplementary Figure S5. Cumulative meta-analysis plot for the risk of VAP for Chlorhexidine + Toothbrush compared to reference interventions.

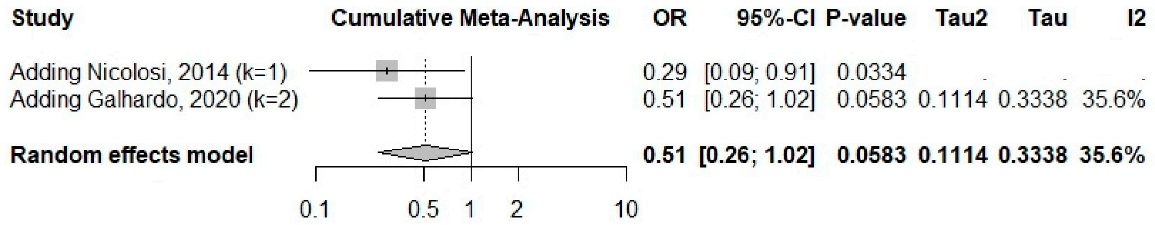

Nicolosi 2014 [80] and Galhardo 2020 [42].

Electronic Supplementary Figure S6. Cumulative meta-analysis plot for the risk of VAP for povidone iodine compared to reference interventions.

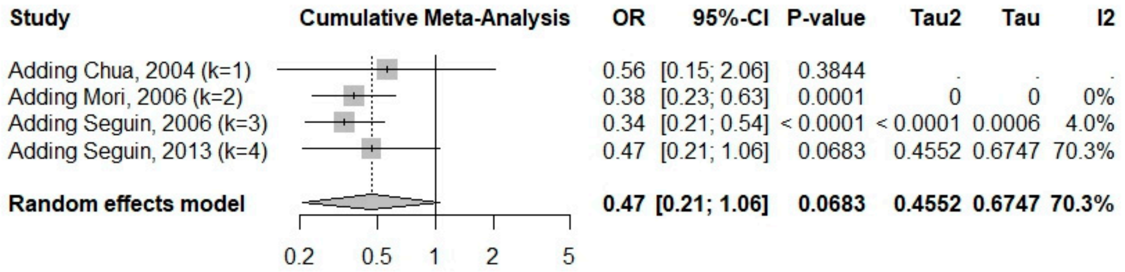

Chua 2004 [29], Mori 2006 [78], Seguin 2006 [95] and Seguine 2013 [96].

Electronic Supplementary Figure S7. Trial sequential analysis for the risk of VAP for chlorhexidine compared to reference interventions.

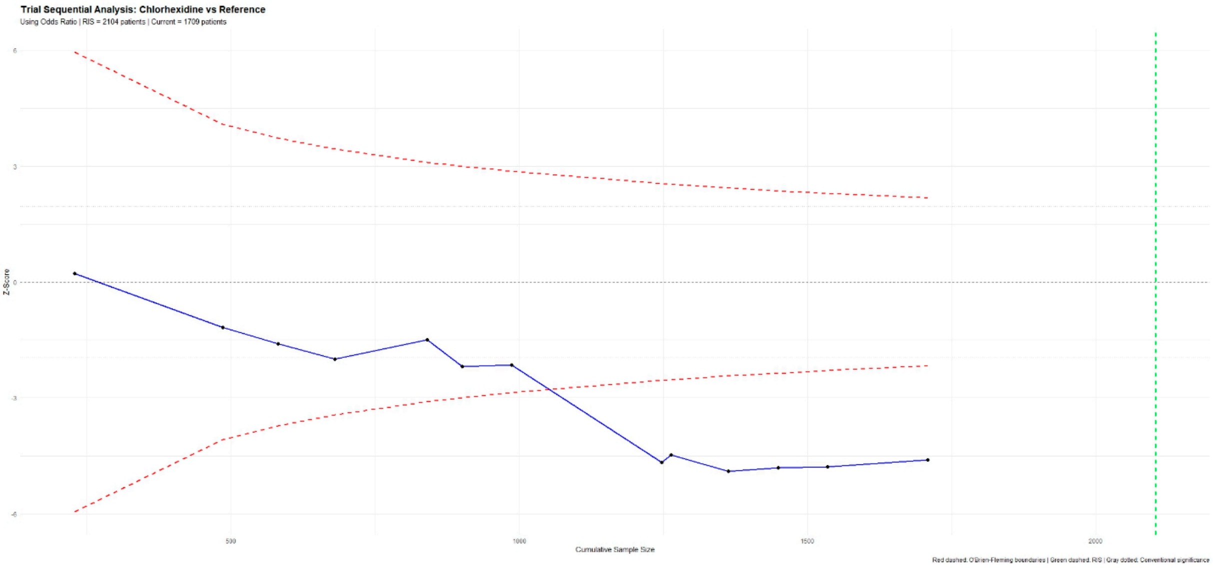

This figure presents the trial sequential analysis for the comparison of chlorhexidine versus reference interventions concerning the risk of VAP.

Electronic Supplementary Figure S8. Trial sequential analysis for the risk of VAP for antimicrobial combination compared to reference interventions.

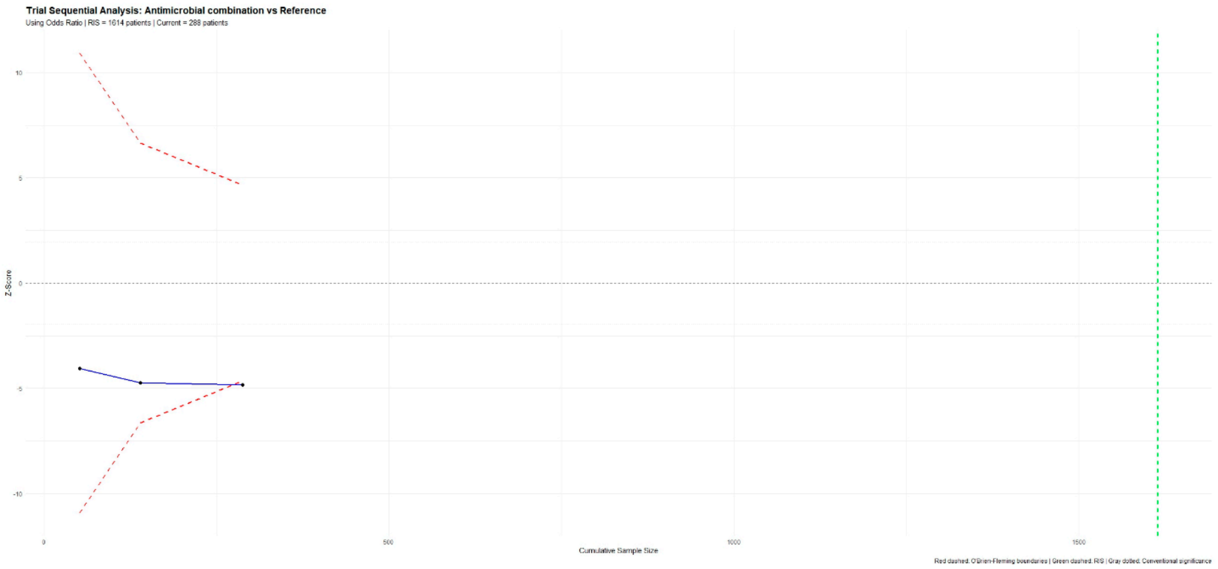

This figure presents the trial sequential analysis for the comparison of antimicrobial combination versus reference interventions concerning the risk of VAP.

Electronic Supplementary Figure S9. Trial sequential analysis for the risk of VAP for povidone iodine compared to reference interventions.

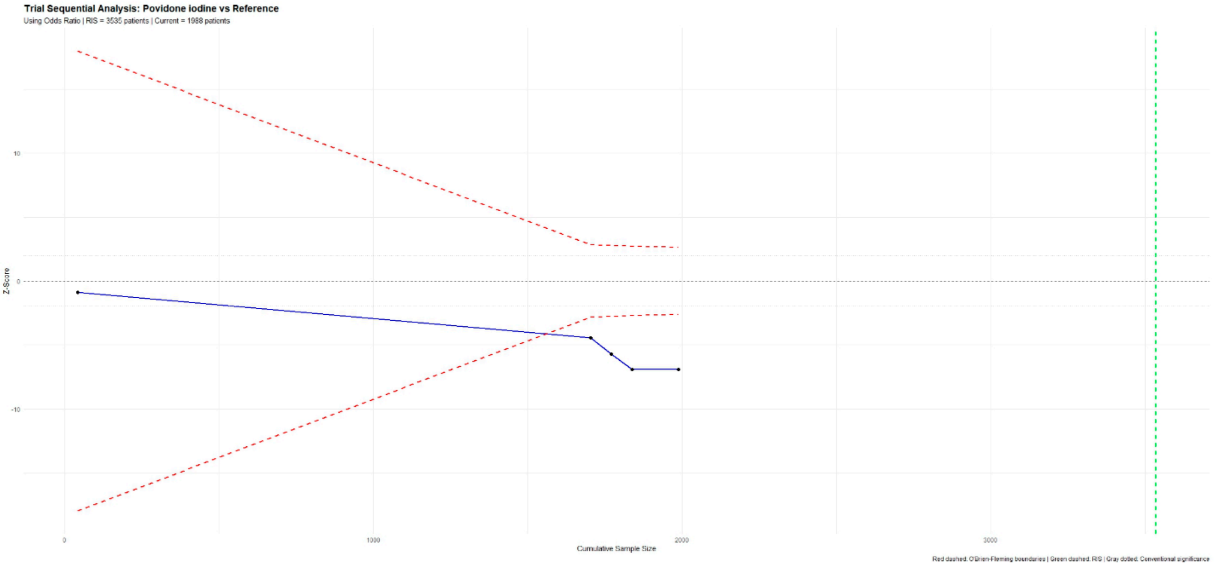

This figure presents the trial sequential analysis for the comparison of povidone iodine versus reference interventions concerning the risk of VAP.
